# Assessing the COVID-19 vaccination program during the Omicron variant (B.1.1.529) epidemic in early 2022, Tokyo

**DOI:** 10.1186/s12879-023-08748-1

**Published:** 2023-10-31

**Authors:** Taishi Kayano, Hiroshi Nishiura

**Affiliations:** Kyoto University School of Public Health, Yoshida-Konoe-cho, Sakyo-ku, Kyoto, 606-8501 Japan

**Keywords:** Immunization, COVID-19, Direct effect, Indirect effect, Population-level impact

## Abstract

**Background:**

Many countries, including high-income nations, struggled to control epidemic waves caused by the Omicron variant (B.1.1.529), which had an antigenically distinct evolution. Evaluating the direct and indirect effects of vaccination during the Omicron waves is essential to assess virus control policies. The present study assessed the population impacts of a vaccination program during the sixth wave caused by BA.1 and BA.2 from January to May 2022, in Tokyo.

**Methods:**

We analyzed the primary series and booster vaccination coverages and the confirmed cases stratified by vaccination history. We estimated the number of COVID-19 cases that were directly and indirectly prevented by vaccination. To estimate the direct impact, we used a statistical model that compared risks between unvaccinated and vaccinated individuals. A transmission model employing the renewal process was devised to quantify the total effect, given as the sum of the direct and indirect effects.

**Results:**

Assuming that the reporting coverage of cases was 25%, mass vaccination programs, including primary and booster immunizations, directly averted 640,000 COVID-19 cases (95% confidence interval: 624–655). Furthermore, these programs directly and indirectly prevented 8.5 million infections (95% confidence interval: 8.4–8.6). Hypothetical scenarios indicated that we could have expected a 19% or 7% relative reduction in the number of infections, respectively, compared with the observed number of infections, if the booster coverage had been equivalent to that of the second dose or if coverage among people aged 10–49 years had been 10% higher. If the third dose coverage was smaller and comparable to that of the fourth dose, the total number of infections would have increased by 52% compared with the observed number of infections.

**Conclusions:**

The population benefit of vaccination via direct and indirect effects was substantial, with an estimated 65% reduction in the number of SARS-CoV-2 infections compared with counterfactual (without vaccination) in Tokyo during the sixth wave caused by BA.1 and BA.2.

**Supplementary Information:**

The online version contains supplementary material available at 10.1186/s12879-023-08748-1.

## Introduction

The COVID-19 pandemic has been a global health emergency since its emergence in late 2019 [1, 2]. Immunization programs have been an integral part of the response to this disease, which is caused by infection with severe acute respiratory syndrome coronavirus 2 (SARS-CoV-2) [3–5]. Mass vaccination programs have two critical pathways to reduce population-level risk: direct and indirect effects [6, 7]. The direct effect of vaccination represents the reduction in the risk of infection or severe disease in vaccinated individuals compared with this risk in unvaccinated individuals. The indirect effect, often referred to as the total effect, results from preventing viral spread in the population, and this effect accumulates when vaccines are efficiently distributed among the population. To ensure successful viral control via mass vaccination and to inform public health policy, evaluating the direct and indirect impacts of vaccination programs is critical.

A small number of studies globally have investigated the population-level impact of COVID-19 vaccination [8–10]. For example, a modeling study in New York City, USA showed that the vaccination program reduced the magnitude of the epidemic during the Alpha (B.1.1.7) and Delta (B.1.617) variant waves, suggesting the importance of accelerating vaccine uptake [8]. A study in Austria measured the population impact of a mass vaccination campaign that took place after a large Beta variant (B.1.351) epidemic by comparing two similar districts, but was not a randomized clinical trial [9]. A statistical modeling study conducted in Israel concluded that the booster vaccination program made substantial direct and indirect contributions to reducing the number of infections, severe cases, and deaths during the Delta variant wave [10]. These studies demonstrate the importance of both the direct and indirect impacts of vaccination, and they highlight the potential for mass vaccination to mitigate the disease burden associated with COVID-19. Since the emergence of Omicron (B.1.1.529), including subvariants BA.1 (B.1.1.529.1), BA.2 (B.1.1.529.2), BA.4 (B.1.1.529.4), and BA.5 (B.1.1.529.5), many countries have struggled to control the virus, partly because of its antigenically distinct evolution. Moreover, very few studies have evaluated vaccination programs at the population level during Omicron waves [11].

Controlling COVID-19 epidemics became more challenging with the emergence of the Omicron variant, partly owing to its increased secondary transmission in vaccinated populations compared with Alpha and Delta [12–17]. Evidence suggests that the vaccines were less effective against Omicron than against previously circulating variants [14, 18]. While the individual benefit of vaccination has been well characterized, the population-level impacts of vaccination during Omicron epidemics have yet to be clarified.

**Fig. 1:**
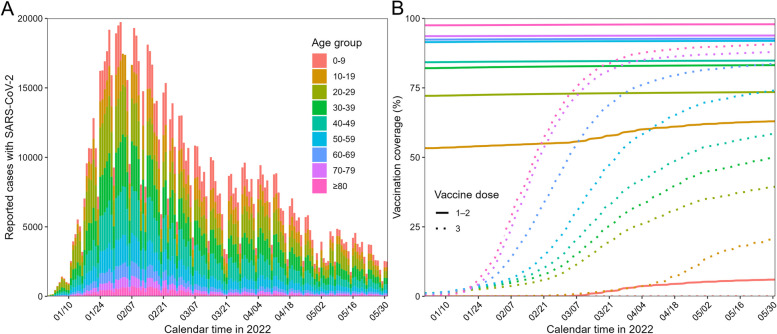
Epidemiology of COVID-19 in Tokyo during the sixth wave, 2022. **A** Number of confirmed SARS-CoV-2 infections from January to May 2022 by age group. Each color represents the number of confirmed cases in each age group. **B** Vaccination coverage stratified by age group and dose (one or three). The period and color of the age group are the same as in Fig. 1A

Community-acquired infections with Omicron BA.1 rose in late December 2021 in Japan, constituting the sixth epidemic wave. During this time, there were approximately 20,000 cases per day in Tokyo. This was the largest COVID-19 epidemic up to that point (Fig. 1A). Shortly before the Omicron epidemic, the booster program had been established in early December 2021, using mainly the Pfizer/BioNTech mRNA vaccine (BNT162b2) and the Moderna vaccine (mRNA-1273); the primary series was also still available (Fig. 1B). In late May 2022, another booster campaign (the fourth dose) was launched initially aiming to cover older people and people with underlying comorbidities. Around this time, the first Omicron BA.5 infections were reported. Epidemiological and vaccination coverage data (Fig. 1) allowed us to reconstruct the transmission dynamics and quantify the population impacts of the vaccination program while focusing on the sixth wave caused by Omicron BA.1 and BA.2.

In the present study, we estimated the population-level impact of vaccination during the Omicron wave. The impact was estimated by distinguishing between the primary series and booster programs.

## Methods

### COVID-19 incidence data

In Japan, all patients diagnosed with COVID-19 at designated healthcare facilities were mandatorily reported to the local public health center in each prefecture under the Infectious Disease Law of 2022. Their personal information including age, sex, and vaccination history was electronically reported via the Health Center Real-time Information-sharing System on COVID-19 (HER-SYS). However, the completeness of the vaccination history information was limited, especially after the surge of Omicron infections in early 2022 [19, 20]. To address this challenge, we focused on the population impact of COVID-19 vaccination in Tokyo, which had more complete vaccination history data, rather than analyzing the impact for the entire country.

To estimate the direct impact, we used the confirmed COVID-19 cases stratified by vaccination history, as reported to the Tokyo metropolitan government. Although there was relatively thorough recordkeeping in Tokyo, the notification data included a small fraction of cases with incomplete vaccination history information (i.e., approximately 25% of cases were not accompanied by vaccination history). We thus performed the subsequent analyses using only complete data by employing a multiple imputation technique. Further explanation of the methods used and a data description can be found in the Additional file. Assuming a consistent delay of 5 days between infection and reporting, the epidemic curve of confirmed cases by the date of confirmation was back-calculated to the curve by the date of infection. The present study analyzed the data from January 1 to May 27, 2022 (21 weeks) during which the Omicron subvariants BA.1 and BA.2 were predominant.

To estimate the total effect, we used the same data stratified by age group. Details of the back-calculation procedure are provided in the Additional file. We estimated the number of infections by age group, assuming that the reporting coverage among all infected individuals was 0.25, i.e., one-quarter of the infections were detected and reported during the study period [21].

### Vaccination coverage

Vaccinated individuals in Japan were registered in a national database called the Vaccine Record System (VRS). We extracted the information (age, vaccination date, and dose number (first or third dose)) of people who were vaccinated in Tokyo between January and May 2022. Because the discrepancy between the coverage of the first and second doses was small (0.6% as of June 1, 2022) according to the VRS, we assumed that all individuals who received a first dose subsequently received a second dose. Thus, our results can be interpreted as estimates for the “primary series” rather than for the first or second dose, specifically. To estimate the direct effect, the vaccination dates were shifted by 14 days into the future to allow for a delay to elicit the immune response [22, 23]. The population immune fractions by age group were then calculated. To compute the total effect, the dataset of vaccinated individuals was converted to that of the immune fraction of the population by using a vaccine efficacy estimate (see Additional file). The vaccinated individuals were divided into nine age groups: 0–9, 10–19, 20–29, 30–39, 40–49, 50–59, 60–69, 70–79, and ≥80 years; the incidence data were divided likewise. Lastly, we imposed a simplifying assumption that vaccine efficacy was independent of age.

### Direct effect

The direct impact was calculated by comparing the risks between vaccinated and unvaccinated individuals. We estimated the total number of averted COVID-19 cases attributable to the direct effect by age group and by vaccine dose, i.e., the primary series or the booster program. The calculation was based on a statistical model whereby the immune fraction was multiplied by the weekly difference in incidence between unvaccinated and vaccinated people, i.e., the risk reduction directly attributable to vaccination, as explained elsewhere [24, 25]. To account for the reporting coverage, the estimates were multiplied by a factor of four to allow for comparison with the population-level impact, as noted above. Uncertainty in the estimates was based on iterations of multiple imputation; thus, the uncertainty reflects variation in the missing values rather than variation in the cases behind the epidemics. Further explanation of the method used is available in the Additional file.

### Total effect

The total effect at the population level, consisting of the vaccine-induced protection that is conferred directly and indirectly, was evaluated by comparing the observed real-world data with a counterfactual scenario in which no vaccination program took place. To do this, we devised a transmission model that reconstructs the transmission dynamics over the period of analysis. A renewal equation was used, and the time-varying transmission model consisted of the incidence history, the effective reproduction number (i.e., the average number of infected cases generated by a single primary case at a given time), and the generation time. The effective reproduction number was expressed as a time-varying matrix that included the immune fraction attributable to the vaccination program, the reduced susceptible fraction owing to natural infection, the social contact matrix, and a weekly scaling parameter. Using the parameterized model and eliminating the vaccination impact (i.e., the immune fraction owing to vaccination) from the fitted transmission model allowed us to produce the counterfactual scenario in which the vaccination program had not taken place. Maximum likelihood estimation was performed to estimate the model parameters assuming that the daily incidence followed a Poisson distribution. The indirect effect was calculated as the gap between the total and direct effects. The 95% confidence intervals (CIs) for the total effectiveness were based on the parametric bootstrap method. We also assessed the impact of a third vaccine dose at the population level by varying the recipients and the coverage of the booster program as different counterfactual scenarios (Additional file).

## Results

The total numbers of prevented COVID-19 cases directly attributable to vaccination by age group and vaccine dose in Tokyo, Japan, from January 1 to May 27, 2022, are shown in Table 1. These estimates were calculated using the confirmed case count; thus, the actual number of directly averted infections is greater. The absolute number of people who benefited from vaccination was highest for adults aged 30–39 years in the primary series program and ≥80 years in the booster program, with estimates of 86,181 (95% CI: 84,743–87,503) and 37,101 (95% CI: 35,649–38,780) people, respectively. Compared with the observed number of cases, the greatest relative reduction due to the direct effect was seen in people over 80 years of age and was estimated as −72% and −54% for the primary series and the booster program, respectively. The youngest age group (0–9 years old) had the lowest number of cases prevented by the primary series program (603 cases; 95% CI: 602–604), which corresponds to a 3% relative reduction compared with the observed count. Throughout the study period, 1–2 doses and a third dose reduced the total number of cases by 29% and 12%, respectively.

**Table 1:** Total number of COVID-19 cases averted owing to reduced risk in vaccinated individuals

Age group (years)	Vaccine dose^a^	Averted cases (95% confidence interval)	Relative change (%)^b^
0–9	Primary series	603 (602–604)	-0.3
	Booster	-	-
10–19	Primary series	65,755 (65,296–66,307)	-31.0
	Booster	2502 (2480–2532)	-1.7
20–29	Primary series	61,872 (60 935–62,910)	-22.0
	Booster	16,909 (16 737–17,063)	-7.1
30–39	Primary series	86,181 (84,743–87,503)	-29.7
	Booster	25,734 (25,382–26,015)	-11.2
40–49	Primary series	45,851 (44,425–47,165)	-19.6
	Booster	23,027 (22,605–23,441)	-10.9
50–59	Primary series	82,251 (80,529–83,825)	-43.4
	Booster	30,268 (29,656–30,858)	-22.0
60–69	Primary series	30,667 (29,400–31,917)	-38.6
	Booster	14,240 (13,786–14,759)	-22.6
70–79	Primary series	22,417 (21,182–23,432)	-40.8
	Booster	11,800 (11,169–12,389)	-26.6
≥80	Primary series	82,424 (79,777–85,023)	-72.5
	Booster	37,101 (35,649–38,780)	-54.3

^a^Primary series represents the vaccination program for the first and second dose, and booster represents the vaccination program for the third dose

^b^Relative change represents a comparison between the calculated counterfactual number and the observed confirmed cases

The transmission model allowed us to calculate the number of SARS-CoV-2 infections in the scenario in which the vaccination program had not taken place. Table 2 shows the age-dependent number of infections prevented by vaccination; these values represent the total impact of vaccination caused by direct and indirect effects. People aged 40–49 years had the highest number of infections averted, estimated at 1,509,663 (95% CI: 1,496,479–1,524,246) people owing to the primary series vaccination and 1,584,700 (95% CI: 1,567,932–1,601,394) people owing to the booster program. The lowest numbers of infections averted were estimated as 500,105 (95% CI: 494,024–506,749) and 471,383 (95% CI: 464,211–478,384) among those aged 70–79 years owing to the primary series plus booster program and ≥80 years owing to the booster program, respectively. However, the most notable relative change due to the total effect was also seen in people aged ≥70 years, with a relative reduction of approximately 80%. The youngest age group, 0–9 years, was again the least likely to benefit directly and indirectly from the vaccination program, yet a relative reduction of approximately 50% was achieved owing to the combined effect of the primary series and booster program.

**Table 2:** Total number of averted SARS-CoV-2 infections attributable to a vaccination program by age group

Age group (years)	Vaccine dose^a^	Averted cases (95% confidence interval)	Relative change (%)^b^
0–9	Primary series and booster	1,375,249 (1,369,273–1,381,905)	-46.6
	Booster	1,425,061 (1,418,927–1,431,610)	-48.4
10–19	Primary series and booster	1,324,726 (1,317,489–1,331,570)	-55.8
	Booster	1,268,196 (1,261,495–1,274,982)	-53.8
20–29	Primary series and booster	2,264,996 (2,250,867–2,280,054)	-61.2
	Booster	2,411,696 (2,398,716–2,424,445)	-63.5
30–39	Primary series and booster	2,267,999 (2,254,956–2,283,361)	-64.0
	Booster	2,392,823 (2,378,472–2,407,955)	-65.9
40–49	Primary series and booster	2,265,627 (2,252,443–2,280,210)	-66.6
	Booster	2,340,664 (2,323,896–2,357,358)	-67.7
50–59	Primary series and booster	1,530,498 (1,518,872–1,544,057)	-71.9
	Booster	1,649,600 (1,634,595–1,664,511)	-73.9
60–69	Primary series and booster	842,916 (835,277–850,903)	-76.8
	Booster	894,544 (883,907–904,551)	-78.1
70–79	Primary series and booster	630,597 (624,516–637,241)	-79.3
	Booster	634,301 (626,165–642,737)	-79.4
≥80	Primary series and booster	636,801 (631,247–642,524)	-80.3
	Booster	596,883 (589,711–603,884)	-79.0

^a^Primary series and booster represents the combined vaccination programs for the first, second, and third doses, and booster represents the vaccination program for the third dos

^b^Relative change represents a comparison between the estimated counterfactual number of infections and the observed number of infections, considering a reporting coverage of 0.25

**Fig. 2:**
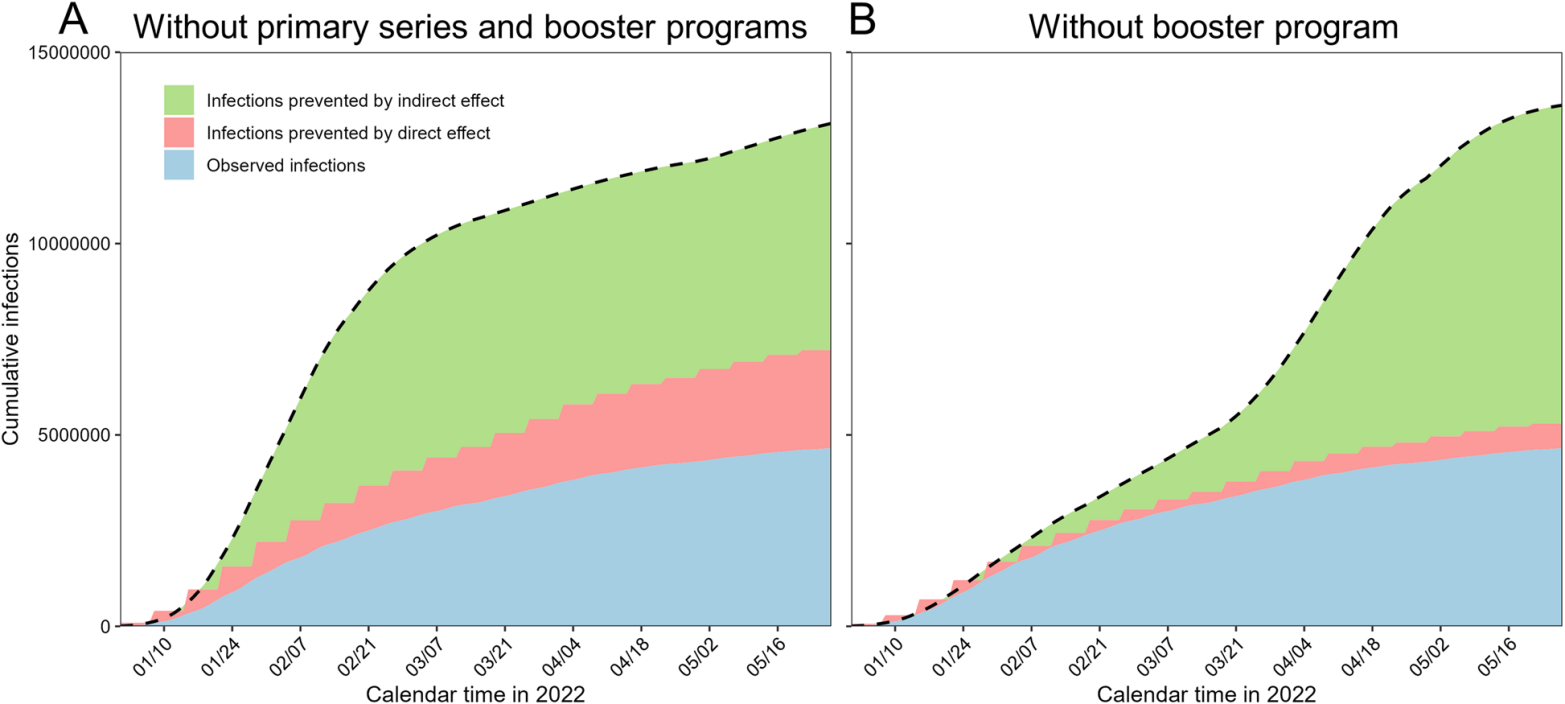
Cumulative number of averted SARS-CoV-2 infections owing to vaccination. The cumulative number of SARS-CoV-2-infected individuals in Tokyo from January 1 to May 27, 2022 in the counterfactual scenario in which (**A**) the primary series and booster programs had not taken place and (**B**) the booster program had not taken place, stratified by type of protection. The blue area represents empirically observed data (confirmed cases divided by an ascertainment bias factor of 25%), the pink area represents infections averted owing to a direct effect, and the green area represents infections averted owing to an indirect effect. The indirect effect was calculated as the gap between the total and direct effects. Dashed lines indicate the cumulative number of infections in the counterfactual scenario with no vaccination program

The population-level impact by vaccine dose is illustrated in Fig. 2. The total impact was estimated at approximately 8.5–9.0 million infections averted by the end of May 2022. The direct effects differed between the programs; 2.6 million infections were prevented by the primary series plus booster program, and 0.6 million infections were prevented by the booster program alone. The indirect impact was obtained by subtracting the direct effect from the total effect, and the proportion of infections indirectly prevented was estimated to be a 70% and 93% total risk reduction owing to the primary series plus booster program and the booster program alone, respectively.

**Fig. 3:**
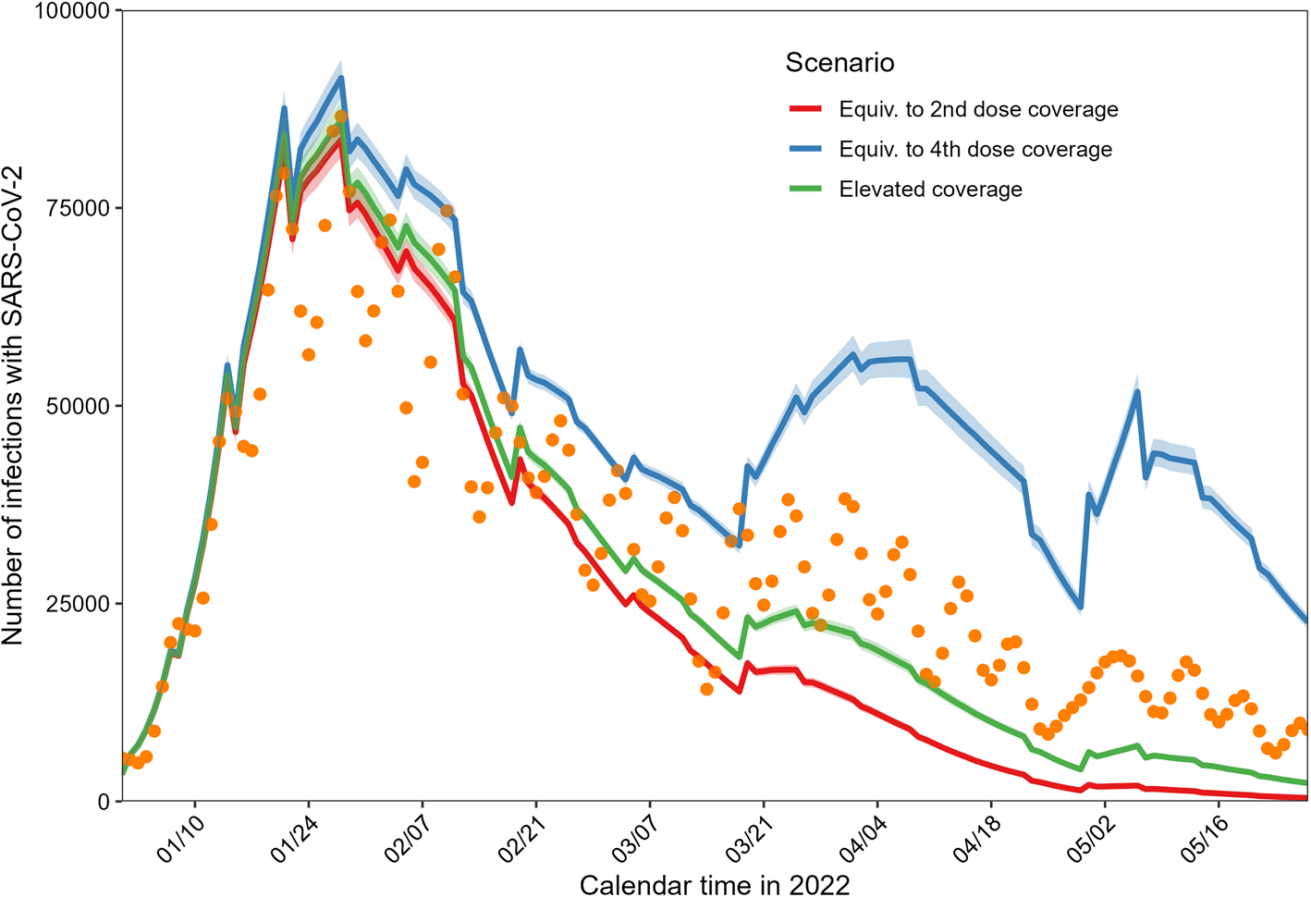
Population-level impact in counterfactual scenarios of booster vaccination. The daily incidence of SARS-CoV-2 infections is shown by counterfactual scenario of booster vaccination in Tokyo. The orange dots represent the empirically observed data (confirmed cases divided by an ascertainment bias factor of 25%). Three scenarios were explored: (i) the vaccination coverage on the last day of the study period (May 27, 2022) was equivalent to that of the primary series (Equiv. to 2nd dose coverage), (ii) the vaccination coverage was equivalent to that of the second booster (i.e., the 4th dose), which was administered later in 2022 (Equiv. to 4th dose coverage), and (iii) the vaccination coverage among people aged 10–49 years was 10% greater than that of the observed third dose (Elevated coverage). Further details can be found in the Additional file

Finally, we explored three possible scenarios of booster dose vaccination by varying the recipients and their coverage (Table 3 and Fig. 3). The details of these counterfactual scenarios are provided in the Additional file. If the booster vaccination coverage was equivalent to that of the fourth dose, we would have experienced a larger epidemic in Tokyo in April and May 2022, reaching a total of 7,084,822 (95% CI: 7,026,286–7,141,322) infections (about half of Tokyo residents). However, if the booster vaccination coverage reached that of the primary series, the number of infections could have been limited to 3,760,075 (95% CI: 3,709,102–3,808,214), a 19% relative reduction compared with the observed number of infections. Moreover, a 10% increase in vaccination coverage among those aged 10–49 years would have reduced the number of infections by 7% by the end of May 2022.

**Table 3:** Cumulative number of SARS-CoV-2 infections in the counterfactual booster scenario

Age group (years)	Booster immunization coverage^a^	Averted cases (95% confidence interval)	Relative change (%)^b^
0–9	Equiv. to 2nd dose	617,501 (610,330–624,323)	-16.0
	Equiv. to 4th dose	983,421 (976,418–990,687)	33.8
	Elevated coverage	690,576 (684,180–696,569)	-6.0
10–19	Equiv. to 2nd dose	477,638 (471,417–483,874)	-18.4
	Equiv. to 4th dose	800,801 (793,776–807,502)	36.8
	Elevated coverage	540,508 (534,463–545,744)	-7.7
20–29	Equiv. to 2nd dose	684,634 (674,962–693,891)	-22.2
	Equiv. to 4th dose	1,344,003 (1,331,788–1,355,746)	52.8
	Elevated coverage	799,613 (791,240–806,938)	-9.1
30–39	Equiv. to 2nd dose	636,277 (627,136–644,993)	-22.1
	Equiv. to 4th dose	1,305,604 (1,293,596–1,317,961)	59.9
	Elevated coverage	747,156 (739,796–754,618)	-8.5
40–49	Equiv. to 2nd dose	608,320 (599,168–616,867)	-19.5
	Equiv. to 4th dose	1,182,119 (1,171,865–1,192,611)	56.4
	Elevated coverage	699,952 (691,723–707,332)	-7.4
50–59	Equiv. to 2nd dose	351,601 (345,798–3,56,991)	-18.3
	Equiv. to 4th dose	714,765 (708,054–721,741)	66.1
	Elevated coverage	412,497 (407,216–417,354)	-4.1
60–69	Equiv. to 2nd dose	164,808 (161,818–167,873)	-15.9
	Equiv. to 4th dose	340,978 (337,381–344,436)	74.0
	Elevated coverage	190,917 (188,132–193,644)	-2.6
70–79	Equiv. to 2nd dose	111,101 (108,918–113,236)	-14.9
	Equiv. to 4th dose	196,119 (194,075–198,270)	50.3
	Elevated coverage	127,683 (125,583–129,707)	-2.2
≥80	Equiv. to 2nd dose	108,193 (106,109–110,355)	-13.8
	Equiv. to 4th dose	217,011 (214,658–219,338)	72.9
	Elevated coverage	125,068 (123,036–127,109)	-0.3

^a^Equiv. to 2nd dose: in the counterfactual booster program scenario, the vaccination coverage was equivalent to that of the second dose; Equiv. to 4th dose: in the counterfactual booster program scenario, the vaccination coverage was equivalent to that of the fourth dose; Elevated coverage: vaccination coverage among people aged 10–49 years was assumed to be 10% higher than the observed coverage

^b^Relative change represents a comparison between the estimated counterfactual number of infections and the observed number of infections, considering a reporting coverage of 0.25

## Discussion

The primary series and booster dose directly contributed to the prevention of 478,000 (95% CI: 467–489) and 162,000 (95% CI: 157–166) COVID-19 cases, representing 29% and 12% relative reductions, respectively, from January to May 2022 in Tokyo. The study period corresponded to the sixth COVID-19 wave in Japan during which the Omicron subvariants BA.1 and BA.2 were predominant. In combination, the primary series plus booster program contributed to directly and indirectly averting 8.5 million (95% CI: 8.4–8.6) SARS-CoV-2 infections. If the booster vaccination coverage had been similar to that of the second dose, or if the coverage among people aged 10–49 years had been 10% greater, the number of infections could have been additionally reduced by 19% and 7%, respectively.

We demonstrated that the population impact of vaccination was substantial in Tokyo even during the epidemics caused by Omicron subvariants BA.1 and BA.2. Overall, the indirect impact accounted for 70% of the total effect (Table S2). The booster dose alone had a smaller direct population impact than the primary series; the booster dose averted 646,000 infections, while the primary series averted 2.6 million infections. From the beginning of the Omicron variant epidemic, vaccine-induced immunity elicited by ancestral virus-based mRNA vaccines was known to have been weaker than that against earlier variants, including Delta. Nevertheless, the vaccination coverage in Japan was high, at greater than 95% for the primary series among older people. The population-level impact was also high even during the Omicron epidemic in 2022, protecting 54% of the population over 60 years of age in Tokyo from infection. Although the sixth wave from January to May 2022 was the largest in Japan by the end of the study period, we found that the population benefited from both direct and, more importantly, indirect protection. Although the Omicron variant was challenging to control, vaccination was a critical public health tool for mitigating COVID-19 [3, 11].

The population-level impact of vaccination was estimated to be greater during the period when the Delta variant predominated, with an estimated 84% relative case reduction in Israel [10], compared with that during the period when Omicron predominated, with an estimated 65% relative case reduction identified in the present study. This difference in impact can be explained by the reduced contribution of the indirect impact rather than the direct impact, because the period of Delta predominance was accompanied by more stringent suppression strategies to control viral transmission and greater vaccine effectiveness compared with the Omicron period [18, 26]. However, the direct impact of the primary series program was still substantial more than a year later, and the booster program elicited additional population impact. This is good news for all populations, especially for those who were previously reluctant to be vaccinated. One of the advantages of studies using mathematical models is that the parameters can be changed, and hypothetical scenarios can be examined [4, 10, 27–29]. As we have shown, a higher vaccination coverage in the population leads to a greater indirect impact at the population level, even in the presence of antigenically distinct evolution, such as the emergence of the Omicron variant, emphasizing that mass vaccination can elicit herd immunity effect even though it may only be temporary.

Our estimates were derived from the sixth COVID-19 wave, which was dominated by Omicron subvariants BA.1 and BA.2; this epidemic was the last wave in Japan in which public health and social measure (PHSM) restrictions were in place. These measures shortened the opening hours of bars and restaurants and aimed to reduce contact in high-risk settings. These measures were in effect in Tokyo from January 21 to March 21, 2022. If the epidemic size had been greater in the absence of PHSMs, the total effect of vaccination would have been even larger. That is, the observed number of cases was affected by the interventions, and in the absence of the PHSMs, the population-level impact would have been larger than estimated.

In the present study, the population impact of vaccination was assessed as the number of averted COVID-19 cases or infections. The analysis could not be extended to include severe cases and deaths because the vaccination history of this population was not thoroughly recorded in any monitoring system in Japan. Considering that vaccination efficiently prevents severe complications in Omicron-infected individuals [3, 18, 30], it would be important to systematically link individual vaccination histories to surveillance or medical record datasets so that an explicit evaluation can be made.

There are several technical limitations to this study. First, estimating the exact number of infections was challenging because symptoms are lessened by vaccine-induced and naturally acquired immunity. The Ministry of Health, Labour, and Welfare conducted seroepidemiological surveys in a serial cross-sectional manner using blood donor data, but the surveys were not conducted regularly throughout the pandemic [31]. We used a reporting coverage of 0.25 as a reference to infer the number of infections during the analysis period in Tokyo [21], and additional sensitivity analyses were performed (see Additional file) with reference to Zhang & Nishiura [32]. Second, we focused on Tokyo because this population had robust data availability, but geographic heterogeneity in the population impact was not assessed. In prefectures with fewer transmissions, a smaller indirect impact might have been observed.

Following our study, additional sublineages (e.g., BA.4, BA.5, BF.7, BQ.1, and XBB) have emerged and have gradually replaced BA.1 and BA.2 partly because of their increased transmissibility, but more importantly because of their immune escape mechanisms. Age-dependent heterogeneity in the immune response has also become recognized, and different sequences of immunization (e.g., primary series vaccination followed by natural infection) have been shown to complicate our understanding of protection at the individual level. However, despite this complexity, the direct and indirect impacts of vaccination can be computed as long as the corresponding vaccination history data are available.

## Conclusions

The primary series and booster vaccination programs prevented many SARS-CoV-2 transmission and contributed to a 65% reduction in infections during the epidemic wave dominated by Omicron BA.1 and BA.2 in Tokyo. Measuring the impact of COVID-19 vaccination can provide valuable information to guide public health policy and improve our understanding of population-level protection. It is critical to achieve high vaccination coverage to benefit from its valuable direct and indirect effects.

### Supplementary Information

**Additional file 1:** **Supplementary Methods.Figure S1.** Vaccine efficacy profile. **Figure S2.** Immune fraction owing to each vaccination program. **Figure S3.** Cumulative number of averted COVID-19 cases owing to the direct effect of the vaccination program. **Figure S4.** Total number of averted COVID-19 cases. **Figure S5.** Proportion of confirmed cases with unknown vaccination history. **Figure S6.** Flowchart of extracting COVID-19 cases for the analysis. **Figure S7.** Prediction accuracy of the deterministic approach to impute missing values for vaccination history. **Figure S8.** Cumulative number of prevented COVID-19 cases based on the deterministic approach. **Figure S9.** Total number of prevented COVID-19 cases based on the deterministic approach. **Figure S10.** Next-generation matrix. **Figure S11.** Cumulative number of SARS-CoV-2 infections: a sensitivity analysis to breakthrough infections.**Figure S12.** Weekly parameter by age group. **Figure S13.** Comparison between observed and predicted SARS-CoV-2 infections by age group. **Figure S14.** Comparison of the total number of observed and predicted SARS-CoV-2 infections assuming a reporting coverage is 0.25. **Figure S15.** Cumulative number of SARS-CoV-2 infections by reporting coverage. **Figure S16.** Comparison between observed and predicted vaccination coverage of the booster program by age group. **Figure S17.** Counterfactual scenarios of the booster vaccination coverage by age group. **Table S1.** Cumulative number of SARS-CoV-2 infections in the absence of vaccination by reporting coverage. **Table S2.** Comparison of population impact of vaccination at the end of the study period (May 27, 2022).

## Data Availability

The data analyzed in this study were publicly available and retrieved from the Tokyo Metropolitan Government, and temporal data of confirmed cases and vaccination coverage not stratified by age group are available at https://stopcovid19.metro.tokyo.lg.jp/.
