# Supplementary material for: Assessing the COVID-19 vaccination program during the Omicron variant (B.1.1.529) epidemic in early 2022, Tokyo
Source: BMC Infect Dis. 2023 Oct 31;23:748. doi: 10.1186/s12879-023-08748-1 (PMC10619277; doi:10.1186/s12879-023-08748-1)
Supplement: Supplementary file 1 — Additional file 1: Supplementary Methods.Figure S1. Vaccine efficacy profile. Figure S2. Immune fraction owing to each vaccination program. Figure S3. Cumulative number of averted COVID-19 cases owing to the direct effect of the vaccination program. Figure S4. Total number of averted COVID-19 cases. Figure S5. Proportion of confirmed cases with unknown vaccination history. Figure S6. Flowchart of extracting COVID-19 cases for the analysis. Figure S7. Prediction accuracy of the deterministic approach to impute missing values for vaccination history. Figure S8. Cumulative number of prevented COVID-19 cases based on the deterministic approach. Figure S9. Total number of prevented COVID-19 cases based on the deterministic approach. Figure S10. Next-generation matrix. Figure S11. Cumulative number of SARS-CoV-2 infections: a sensitivity analysis to breakthrough infections.Figure S12. Weekly parameter by age group. Figure S13. Comparison between observed and predicted SARS-CoV-2 infections by age group. Figure S14. Comparison of the total number of observed and predicted SARS-CoV-2 infections assuming a reporting coverage is 0.25. Figure S15. Cumulative number of SARS-CoV-2 infections by reporting coverage. Figure S16. Comparison between observed and predicted vaccination coverage of the booster program by age group. Figure S17. Counterfactual scenarios of the booster vaccination coverage by age group. Table S1. Cumulative number of SARS-CoV-2 infections in the absence of vaccination by reporting coverage. Table S2. Comparison of population impact of vaccination at the end of the study period (May 27, 2022). [file 12879_2023_8748_MOESM1_ESM.docx]

Additional file 1.

Supplementary Appendix

**Assessing the COVID-19 vaccination program during the Omicron variant (B.1.1.529) epidemic in early 2022, Tokyo**

Taishi Kayano^1^, Hiroshi Nishiura^1^

^1^ Kyoto University School of Public Health, Yoshida-Konoe-cho, Sakyo-ku, Kyoto 606-8501, Japan

**Table of contents**

[Supplementary Methods 2](#_Toc132367807)

[Estimation of the number of infections 2](#_Toc132367808)

[Estimation of the immune fraction 2](#_Toc132367809)

[Statistical model to examine the direct effect 3](#_Toc132367810)

[Multiple imputation to compute the direct impact 4](#_Toc132367811)

[Transmission model to examine the total effect 4](#_Toc132367812)

[Vaccination scenarios 6](#_Toc132367813)

[Supplementary References 7](#_Toc132367814)

[Supplementary Figures 9](#_Toc132367815)

[Supplementary Tables 22](#_Toc132367816)

**Supplementary Methods**

We devised a statistical model and transmission model to estimate the numbers of COVID-19 cases and infections, respectively, that were averted in Tokyo from January to May 2022, owing to the vaccination program. The following sections provide detailed information, including links, on the methodology of our research. Additional analyses are also presented with tables and figures, including a sensitivity analysis.

**Estimation of the number of infections**

To estimate the direct effect of vaccination, all confirmed cases in Tokyo reported to the Tokyo metropolitan government from January to June 2022 were back-calculated assuming a 5-day time delay between infection and reporting, estimated from registered cases in Tokyo at the Health Center Real-time Information-sharing System on COVID-19 (HER-SYS). These cases were retrieved based on the estimated infection dates of January 1 to May 27, 2022.

To estimate the total effect, all confirmed cases reported to the Tokyo government between December 25, 2021 and July 31, 2022, were back-calculated to cases at symptom onset using the time between symptom onset and confirmation, which was assumed to follow a log-normal distribution with a mean of 2.1 days and a standard deviation (SD) of 1.9 days. The distribution was estimated from the HER-SYS data. Next, those cases were back-calculated to infected individuals using a distribution of the interval between infection and disease onset that was assumed to follow a gamma distribution with a mean of 3 days and a SD of 1.5 days [1]. Non-parametric back-calculation was performed using the R-package “surveillance” (version 1.20.3) to estimate the number of infections. As with the direct effect calculations, the cases infected during the study period were used in the analysis.

It must be noted that back-calculation methods were different between direct and total effects, because the data sources and associated methods were fairly different, i.e., direct effect required the incidence data by vaccination history and the computation using those datasets alone, while total effects called for an estimation of the effective reproduction number from the incidence of infection. Thus, to be strict, comparison of those effects in real time might not be technically supported, but at least we ensured that the mean time delay from infection to reporting (or confirmation) was 5 days.

**Estimation of the immune fraction**

Using the Vaccine Record System (VRS), we estimated the immune fraction stratified by age group and dose in Tokyo, taking into account waning immunity. First, individuals vaccinated in Tokyo during the study period were retrieved from the VRS according to vaccination date. Because the discrepancy in vaccination coverage between the first dose (77.11%) and the second dose (76.50%) was small in Japan as of June 1, 2022 [2], we assumed that those who received the first dose were subsequently vaccinated with the second dose. Therefore, we used the information of those vaccinated with the first and third dose to represent the populations who completed the primary series and the booster program, respectively.

To estimate the direct effect, we added 14 days to the vaccination date to obtain the time-dependent increase in immunized people on specific days by age group for the statistical model that estimated the effect. Thus, we assumed that the vaccine was ineffective for the first 14 days after vaccination, followed by an abrupt increase in vaccine-induced immunity thereafter; the third dose was treated the same way [3, 4]. We finally obtained the vaccination coverage, which took into account the build-up and waning of immunity, by dividing the cumulative number of people immunized by the population size.

We used the transmission model to estimate the total effect. We used vaccine efficacy profiles against symptomatic infection for those who completed the primary series and the booster vaccination program to estimate the immune fraction. First, using the previous estimates of vaccine efficacy against symptomatic infection, we developed the profile, assuming exponential decay, for vaccinated individuals in each program. Second, we assumed that all individuals who received the first dose were subsequently vaccinated with the second dose at a 21-day interval.

For the estimation of total effect, we assumed that the time required to reach peak vaccine efficacy was 21 days since first dose. The vaccine efficacy profile with waning immunity for the primary series is shown in Figure S1A, which was based on a previous estimate [5]. The peak protection occurs around 21 days after vaccination, because we estimated the efficacy profile from a published dataset with discrete interval of time since first dose, and the mid-point of peak efficacy was 21 days since first dose. For the booster program, it took 21 days to reach maximum vaccine efficacy and then declined exponentially (Figure S1B), for exactly the same reason [5]. Both vaccine efficacy estimates were based on the messenger RNA vaccine BNT162b2 (Pfizer/BioNTech), and we believe they apply to the Japanese situation, because 80% of residents had received this vaccine by March 2023 [6]. Finally, to estimate the immune fraction, $J_{a,t,k}$, in age group $a$ owing to vaccination program $k$ (the primary series or booster program) at calendar time $t$, the following equation was employed:

| $J_{a,k,t}=\frac{1}{n_{a}}\sum_{s=1}^{t-1} v_{a,k,t-s}e_{s,k},$ | (1) |
| --- | --- |

where $n_{a}$ is the population size in age group $a$ in Tokyo in 2022 [7], $v_{a,t}$ represents the number of vaccinated individuals in age group $a$ for vaccination program $k$ at time $t$ retrieved from the VRS, and $e_{t,k}$ denotes the vaccine efficacy profile of vaccination program $k$. Figure S2 shows the immune fraction by age group and vaccination program. Using the estimated immune landscape similar to ours, a published study took place to evaluate the population impact of vaccination [8].

**Statistical model to examine the direct effect**

The direct impact can be calculated by comparing the risks between vaccinated and unvaccinated individuals. After conducting multiple imputation (explained in the following subsection), we estimated the total number of averted cases in age group $a$ that could be attributed to the direct impact of vaccination program $k$ (primary series or booster program) from January 1 to May 27, 2022, $h_{a,k}$, described as:

| $h_{a,k}=\sum_{w=1}^{21} n_{a}l_{a,k,w}(r_{a,unvac,w}-r_{a,k,w}),$ | (2) |
| --- | --- |

where $l_{a,k,w}$ is the vaccination coverage (taking into account the process of building and waning immunity) of program $k$ in age group $a$ in week $w$, and $r_{a,unvac,w}-r_{a,k,w}$ represents the weekly incidence difference, i.e., the risk reduction directly attributable to the vaccination program, as explained elsewhere [9, 10]. The cumulative number of prevented cases by age group in each vaccination program is shown in Figure S3. The total number of averted cases by vaccination program (i.e., primary series or booster program) is illustrated in Figure S4.

To account for reporting coverage, the estimates were multiplied by four to allow for comparison with the population-level effect (shown in Figure 2 in the main text and Table S1) [11].

**Multiple imputation to compute the direct impact**

We used data from the Tokyo government, which included confirmed cases with information on the confirmation date, age, sex, presence of symptoms, metropolitan district where detected, and vaccination history. To estimate the weekly incidence among unvaccinated people ($r_{a,k,unvac}$) and vaccinated people ($r_{a,k,w}$), we needed the numerator of the weekly incidence, i.e., the weekly number of confirmed cases in age group $a$ according to vaccination status (primary series: 1st and 2nd doses or booster program: 3rd dose). This meant that the vaccination history was necessary for the analysis. However, the vaccination history data contained missing values, which was almost constant at approximately 25% over the study period (Figure S5). To complete the missing information, we employed multiple imputation based on fully conditional specification using the ‘mice’ package (version 3.15.0) in R [12]. To do this, we extracted the cases with complete data on age, sex, presence of symptoms, and metropolitan district where detected for the multiple imputation; a detailed flowchart is provided in Figure S6. We performed the multiple imputation 1,000 times to infer the uncertainty of the direct effect estimation. Thus, the 95% confidence intervals (CIs) for the estimates of the direct impact were based on the distributions of missing information rather than on those of confirmed cases, which reflected the dynamics of the epidemic.

In addition, we used a deterministic approach to impute the missing information to estimate the direct impact of the vaccination program using multinominal logistic regression as a sensitivity analysis. The prediction accuracy, calculated as the total number of correctly predicted vaccination statuses divided by the total number of predicted cases, for the already known values is shown in Figure S7. The cumulative number of averted cases by age group and total number of prevented cases by vaccination program are illustrated in Figures S8 and S9, respectively.

**Transmission model to examine the total effect**

The total effect of vaccination at the population level can be measured by comparing the number of observed infections with the number in the counterfactual scenario in which vaccination had not taken place. For this purpose, we devised a transmission model that reconstructed the transmission dynamics over the course of the analysis period. The following equation using the renewal process was employed:

| $i_{a,t}=\sum_{b=1}^{9} \sum_{\tau=1}^{t-1} \boldsymbol{R}_{\boldsymbol{ab}\boldsymbol{,}\boldsymbol{t}}i_{b,t-\tau}g_{\tau},$ | (3) |
| --- | --- |

where $i_{a,t}$ is the daily number of SARS-CoV-2 infections in age group $a$ at time $t$, $\boldsymbol{R}_{\boldsymbol{ab,t}}$ represents the effective reproduction number, i.e., the average number of infections in age group $a$ generated by a single primary case in age group $b$, and $g_{\tau}$ represents the generation time [13]. $\boldsymbol{R}_{\boldsymbol{ab,t}}$ is expressed as a time-varying matrix consisting of the following parameters:

| $\boldsymbol{R}_{\boldsymbol{ab}\boldsymbol{,}\boldsymbol{t}}=q_{a,w}(1-(J_{a,1-2,t}+J_{a,3,t}+J_{a,t}^{infection}))\boldsymbol{m}_{\boldsymbol{ab}},$ | (4) |
| --- | --- |

where $q_{a,w}$ is the weekly scaling parameter in age group $a$ in week $w$, $J_{a,k,t}$ represents the immune fraction in age group $a$ at calendar time $t$ attributable to vaccination program $k$ (primary series: the first and second doses or booster program: the third dose), $J_{a,t}^{infection}$ represents the immune fraction owing to immunity that is naturally acquired from infection, and $\boldsymbol{m}_{\boldsymbol{ab}}$ is a next-generation matrix. $\boldsymbol{m}_{\boldsymbol{ab}}$ was based on the social contact matrix [14] and relative susceptibility in age group $a$, which was quantified using data from the Alpha variant epidemic in Osaka, Japan in March–April 2021. The comparison between the observed and predicted values estimated from the model and the quantified matrix are shown in Figure S10, and a detailed explanation is available elsewhere [15]. Eliminating the vaccination effect from the fitted transmission model allowed us to produce the counterfactual scenario in which the vaccination program had not taken place. It should be noted that $J_{a,k,t}$ and $J_{a,t}^{infection}$ are independently subtracted from 1, and this calculation reflects an assumption that the vaccine-induced immunity is independent from the acquired immunity from natural infection. We imposed this strong assumption, because the size of first Omicron epidemic wave was yet very small (i.e. less than 5% of the population with natural infection via anti-nucleocapsid antibody [16]) and breakthrough events were yet limited in late 2022 (e.g. 1.2% of confirmed cases [17]). However, to address possible involvement of many breakthrough events, we carried out sensitivity analysis, by using the following alternative equation, i.e.,

| $\boldsymbol{R}_{\boldsymbol{ab}\boldsymbol{,}\boldsymbol{t}}=q_{a,w}(1-(J_{a,1-2,t}+J_{a,3,t}+(1-\rho)J_{a,t}^{infection}))\boldsymbol{m}_{\boldsymbol{ab}},$ | (5) |
| --- | --- |

where the fraction $\rho$ represents the history of past vaccination among naturally infected individuals. That is, by varying the value of $\rho$ and estimating the total effect, we can examine the sensitivity of the total effect to breakthrough infection dynamics. Figure S11 shows the corresponding result from sensitivity analysis. By the end of research period, the cumulative number of infections without vaccination (counterfactual scenario) reached approximately 14.3 ($\rho=0.1$), 15.8 ($\rho=0.2$), 17.8 ($\rho=0.3$), and 20.4 hundred thousand ($\rho=0.4$), respectively. That is, as the frequency of breakthrough events increased, the estimated total effect was magnified, and our baseline results in the main text ($\rho=0$) is a potential underestimate of the total effect of vaccination.

Maximum likelihood estimation was performed to estimate the unknown parameter, $q_{a,w}$, assuming that the daily incidence followed a Poisson distribution, which was represented as:

| $L\left( q_{a,w};i_{a,t} \right)=\prod_{t} \prod_{a} \frac{{E(i_{a,t})}^{i_{a,t}}exp(-E(i_{a,t}))}{i_{a,t}!}.$ | (6) |
| --- | --- |

We estimated $q_{a,w}$ by minimizing the loglikelihood function of Eq. (6). The 95% CIs of the total vaccination effect were based on the parametric bootstrap method with 1,000 bootstrap iterations using the multivariate normal distributions of the parameters. We estimated the parameters by reporting the coverage, although we generally assumed a reporting coverage of 0.25. The estimated weekly parameters by age group are shown in Figure S12 and assume a reporting coverage of 0.25. A comparison of observed and predicted infections by age group is shown in Figure S13. Table S2 shows the comparison of the cumulative number of infections by the population-level impact owing to the vaccination program. We also conducted a sensitivity analysis to explore the variation in reporting coverage (i.e., 0.125, 0.25, or 0.5), and a comparison of the total number of infections by reporting coverage is provided in Figure S14. A comparison of the cumulative number of infections between the real-world situation and the counterfactual scenario (in which no vaccination program took place) by reporting coverage is shown in Figure S15.

In the present study, we assumed that people infected with SARS-CoV-2 experienced contracting immunity following the same profile as in the primary series vaccination program. Individuals infected before the study period were not considered because less than 3% of the population of Tokyo was infected by the end of 2021 (although, assuming a reporting coverage of 0.25, 10% of the population had been infected) [18]. In addition, we assumed that children younger than 10 had not been vaccinated; therefore, their impact was not explored. For the initial conditions, we used back-calculated infections from December 25 to December 31, 2021, to examine the fitted transmission model because data were only available for a limited period.

**Vaccination scenarios**

In addition to a no-vaccination scenario, using the estimated parameter $q$, we also explored hypothetical scenarios by varying the timing and the recipients of the booster vaccination program. For convenience, we labeled the scenarios in which vaccination coverage had reached the level equivalent to the second dose on December 31, 2021, equivalent to the fourth dose on December 31, 2022, and increased by 10% in the 10–49 age group as “Equiv. to 2nd dose,” “Equiv. to 4th dose,” and “Elevated coverage,” respectively. To assess the counterfactual scenarios, we regressed the booster program vaccination coverage on the following logistic function by age group:

| $E(v_{a,t})=\frac{\alpha_{a}}{1+exp(-\beta_{a}(t-\gamma_{a}))},$ | (7) |
| --- | --- |

where $\alpha_{a}$, $\beta_{a}$, and $\gamma_{a}$ represent the age-dependent parameters that characterize vaccination coverage. $\alpha_{a}$ represents the carrying capacity (i.e., the eventual coverage of the primary series at time $\infty$), $\beta_{a}$ represents the rate (speed) of increase in the vaccination coverage, and $\gamma_{a}$ denotes the time required for vaccination coverage to reach half of $\alpha_{a}$, respectively. We used maximum likelihood estimation to estimate $\alpha_{a}$, $\beta_{a}$, and $\gamma_{a}$ by age group. Comparisons between the predicted and observed number of people vaccinated by age group are shown in Figure S16. Figure S17 shows all counterfactual scenarios and the baseline scenario.

**Supplementary References**

1. National Institute of Infectious Diseases. Estimation of the incubation period of mutant strain B.1.1.529 (Omicron strain) of SARS-CoV-2: a preliminary report. https://www.niid.go.jp/niid/ja/2019-ncov/2551-cepr/10903-b11529-period.html. Accessed 22 Mar 2023.

2. Digital Agency of Japan. Digital Agency of Japan. https://info.vrs.digital.go.jp/dashboard/. Accessed 20 Mar 2023.

3. Polack FP, Thomas SJ, Kitchin N, Absalon J, Gurtman A, Lockhart S, et al. Safety and Efficacy of the BNT162b2 mRNA Covid-19 Vaccine. New England Journal of Medicine. 2020;383:2603–15.

4. Dagan N, Barda N, Kepten E, Miron O, Perchik S, Katz MA, et al. BNT162b2 mRNA Covid-19 Vaccine in a Nationwide Mass Vaccination Setting. New England Journal of Medicine. 2021;384:1412–23.

5. UK Health Security Agency. COVID-19 vaccine surveillance report: week 48. 2022.

6. Prime Minister’s Office of Japan. Daily achievements: COVID-19 vaccine. https://www.kantei.go.jp/jp/headline/kansensho/vaccine.html. Accessed 23 Mar 2023.

7. Statistics Bureau: Ministry of Internal Affairs and Communications Japan. Summary of population estimates. https://www.e-stat.go.jp/stat-search?page=1&query=%E4%BA%BA%E5%8F%A3&layout=normal. Accessed 23 Mar 2023.

8. Gavish N, Yaari R, Huppert A, Katriel G. Population-level implications of the Israeli booster campaign to curtail COVID-19 resurgence. Sci Transl Med. 2022;14:9836.

9. Kayano T, Sasanami M, Kobayashi T, Ko YK, Otani K, Suzuki M, et al. Number of averted COVID-19 cases and deaths attributable to reduced risk in vaccinated individuals in Japan. Lancet Reg Health West Pac. 2022;28.

10. Haas EJ, McLaughlin JM, Khan F, Angulo FJ, Anis E, Lipsitch M, et al. Infections, hospitalisations, and deaths averted via a nationwide vaccination campaign using the Pfizer-BioNTech BNT162b2 mRNA COVID-19 vaccine in Israel: a retrospective surveillance study. Lancet Infect Dis. 2022;22:357–66.

11. Sanada T, Honda T, Yasui F, Yamaji K, Munakata T, Yamamoto N, et al. Serologic Survey of IgG Against SARS-CoV-2 Among Hospital Visitors Without a History of SARS-CoV-2 Infection in Tokyo, 2020-2021. J Epidemiol. 2022;32:105–11.

12. Liu Y, De A. Multiple Imputation by Fully Conditional Specification for Dealing with Missing Data in a Large Epidemiologic Study HHS Public Access. Int J Stat Med Res. 2015;4:287–95.

13. Backer JA, Eggink D, Andeweg SP, Veldhuijzen IK, van Maarseveen N, Vermaas K, et al. Shorter serial intervals in SARS-CoV-2 cases with Omicron BA.1 variant compared with Delta variant, the Netherlands, 13 to 26 December 2021. Eurosurveillance. 2022;27:2200042.

14. Munasinghe L, Asai Y, Nishiura H. Quantifying heterogeneous contact patterns in Japan: a social contact survey. Theor Biol Med Model. 2019;16:1–10.

15. Sasanami M, Kayano T, Nishiura H. Monitoring the COVID-19 immune landscape in Japan. International Journal of Infectious Diseases. 2022;122:300–6.

16. National Institute of Infectious Diseases. Report on seroepidemiological surveillance for COVID-19 in 2021. https://www.niid.go.jp/niid/ja/2019-ncov/2484-idsc/11118-covid19-79.html. Accessed 22 Mar 2023.

17. Kitamura N, Otani K, Kinoshita R, Yan F, Takizawa Y, Fukushima K, et al. Protective effect of previous infection and vaccination against reinfection with BA.5 Omicron subvariant: a nationwide population-based study in Japan. Lancet Reg Health West Pac. 2023;0:100911.

18. Tokyo Metropolitan Government. Trends in the number of positive cases of COVID-19 by reporting date. https://stopcovid19.metro.tokyo.lg.jp/cards/number-of-confirmed-cases/. Accessed 23 Mar 2023.

**Supplementary Figures**


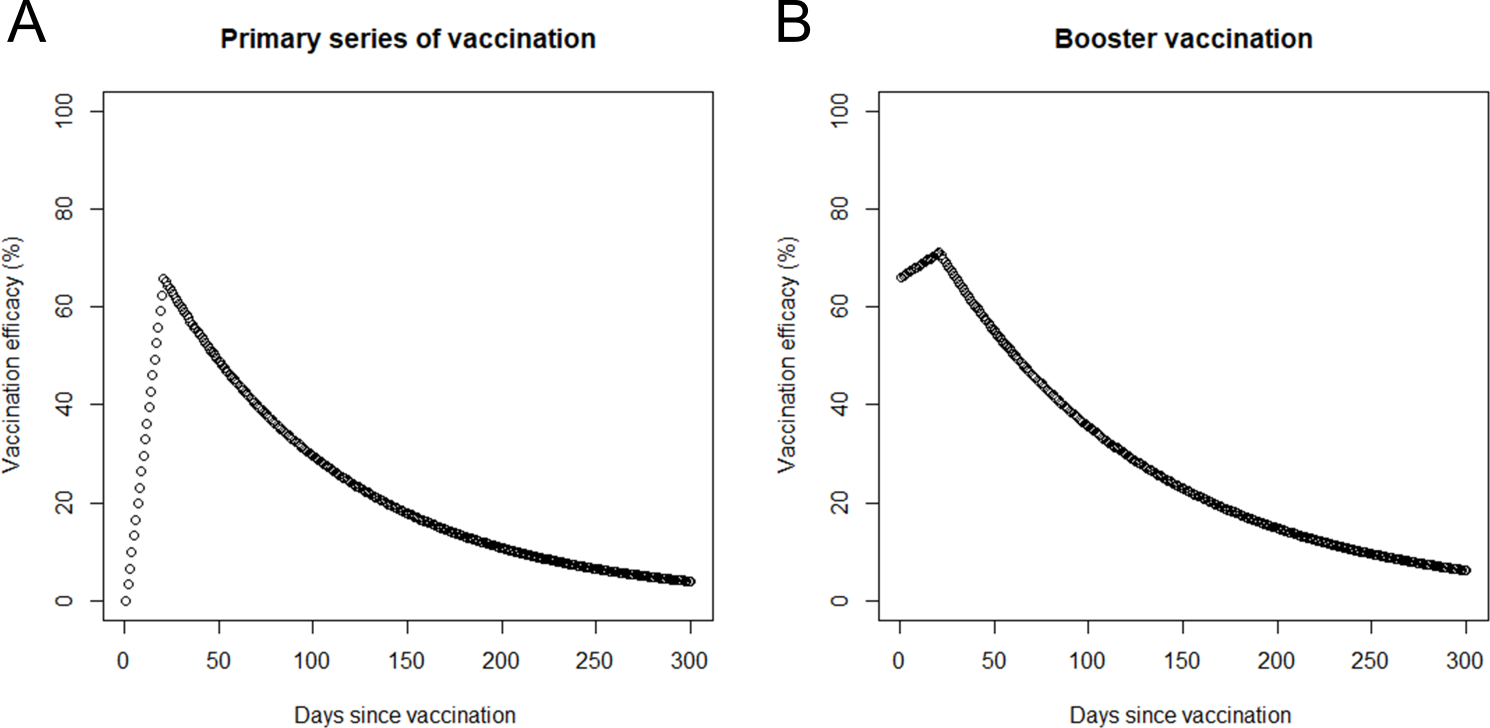


**Figure S1. Vaccine efficacy profile.**

(A) Vaccine efficacy profile used for those who completed the primary series (1st and 2nd doses). (B) Vaccine efficacy profile used for those who received the booster dose, i.e., 3rd dose.


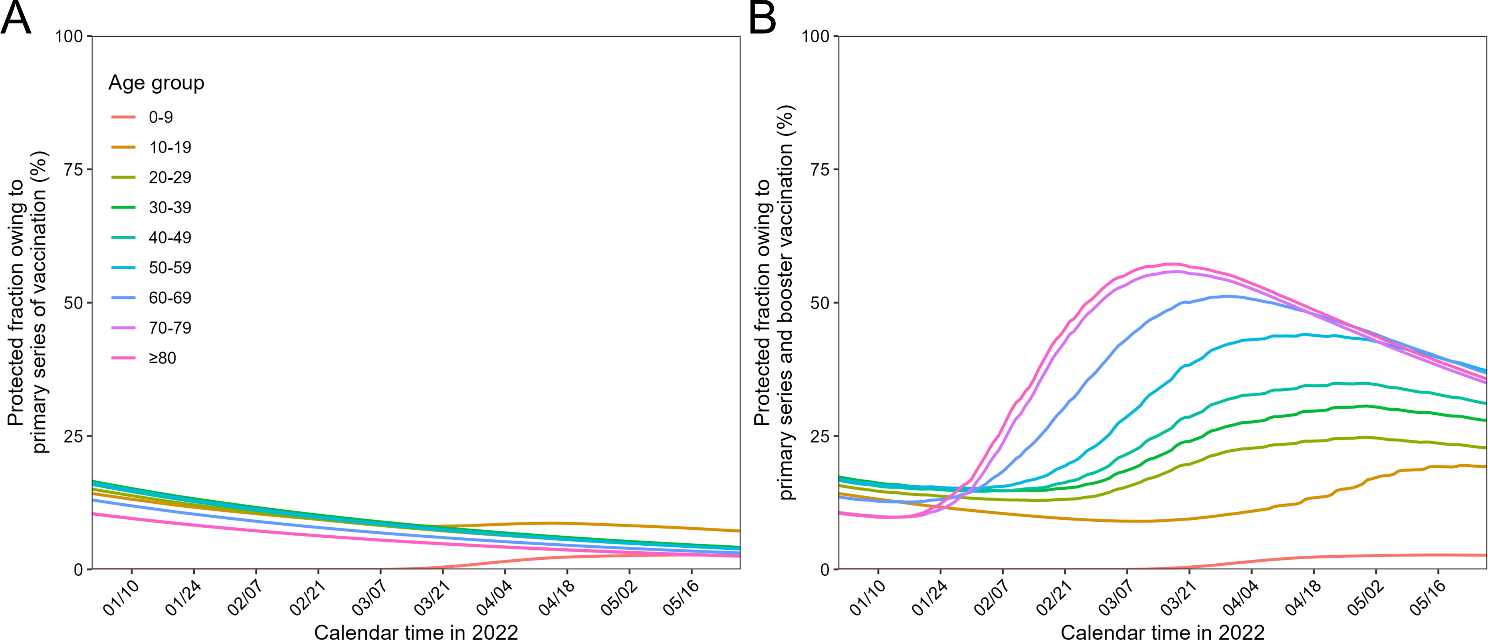


**Figure S2. Immune fraction owing to each vaccination program.**

Fraction protected against SARS-CoV-2 owing to (A) the primary series and (B) the booster vaccination program by age group.


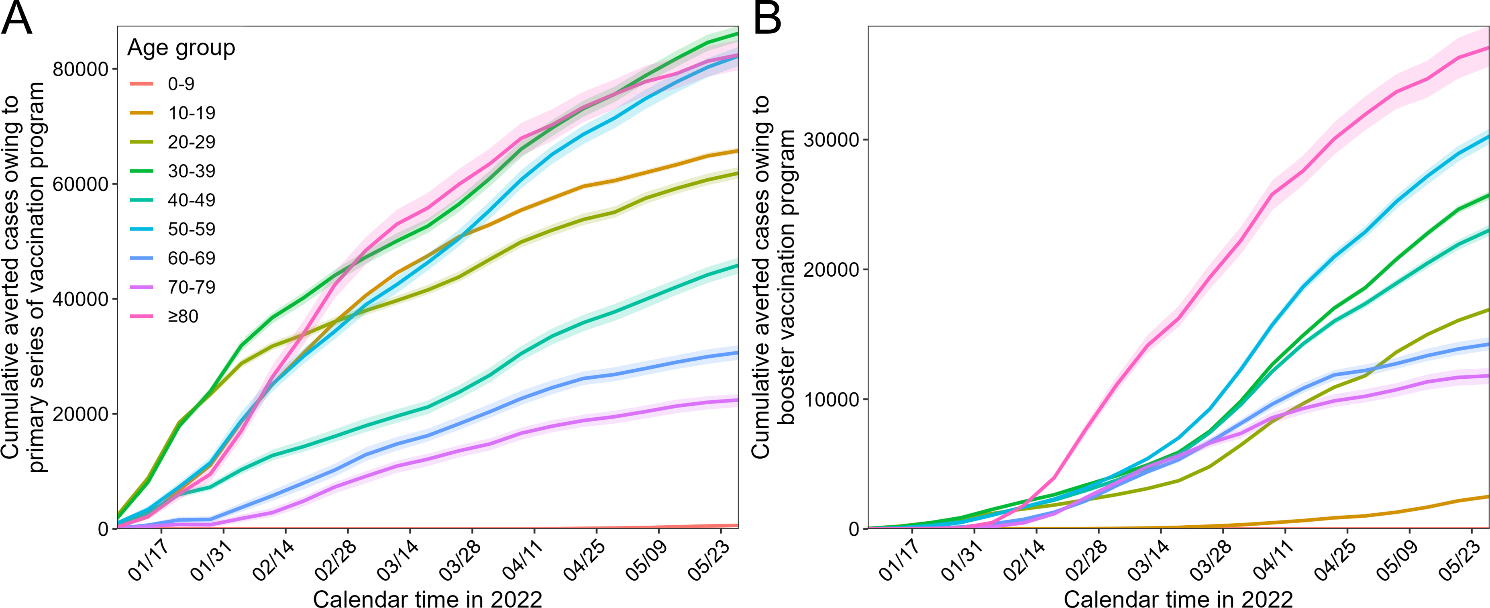


**Figure S3. Cumulative number of averted COVID-19 cases owing to the direct effect of the vaccination program.**

Cumulative number of COVID-19 cases averted that is directly attributable to (A) the primary series and (B) the booster vaccination program by age group. The 95% confidence intervals for each age group are shown in the lighter color.


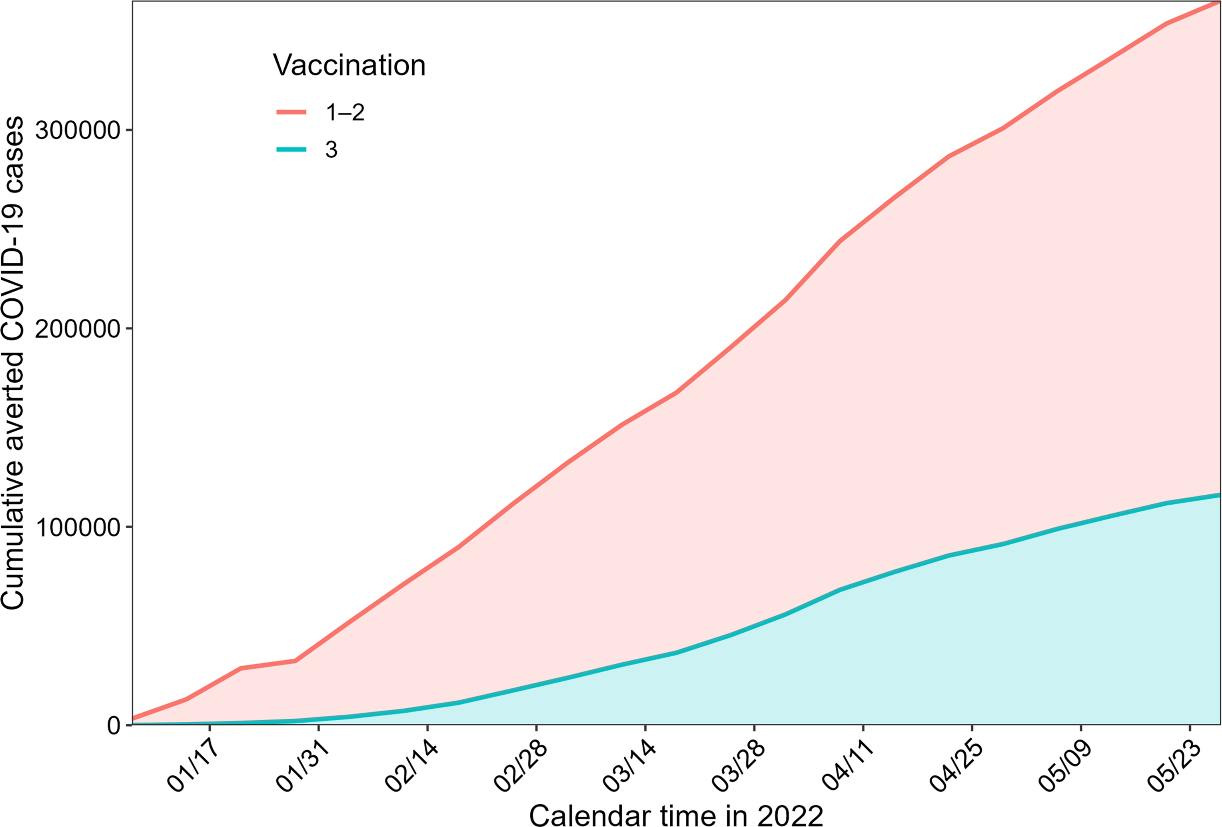


**Figure S4. Total number of averted COVID-19 cases.**

Total numbers of COVID-19 cases averted that are directly attributable to the primary series (1–2 doses) and the booster program (3rd dose).


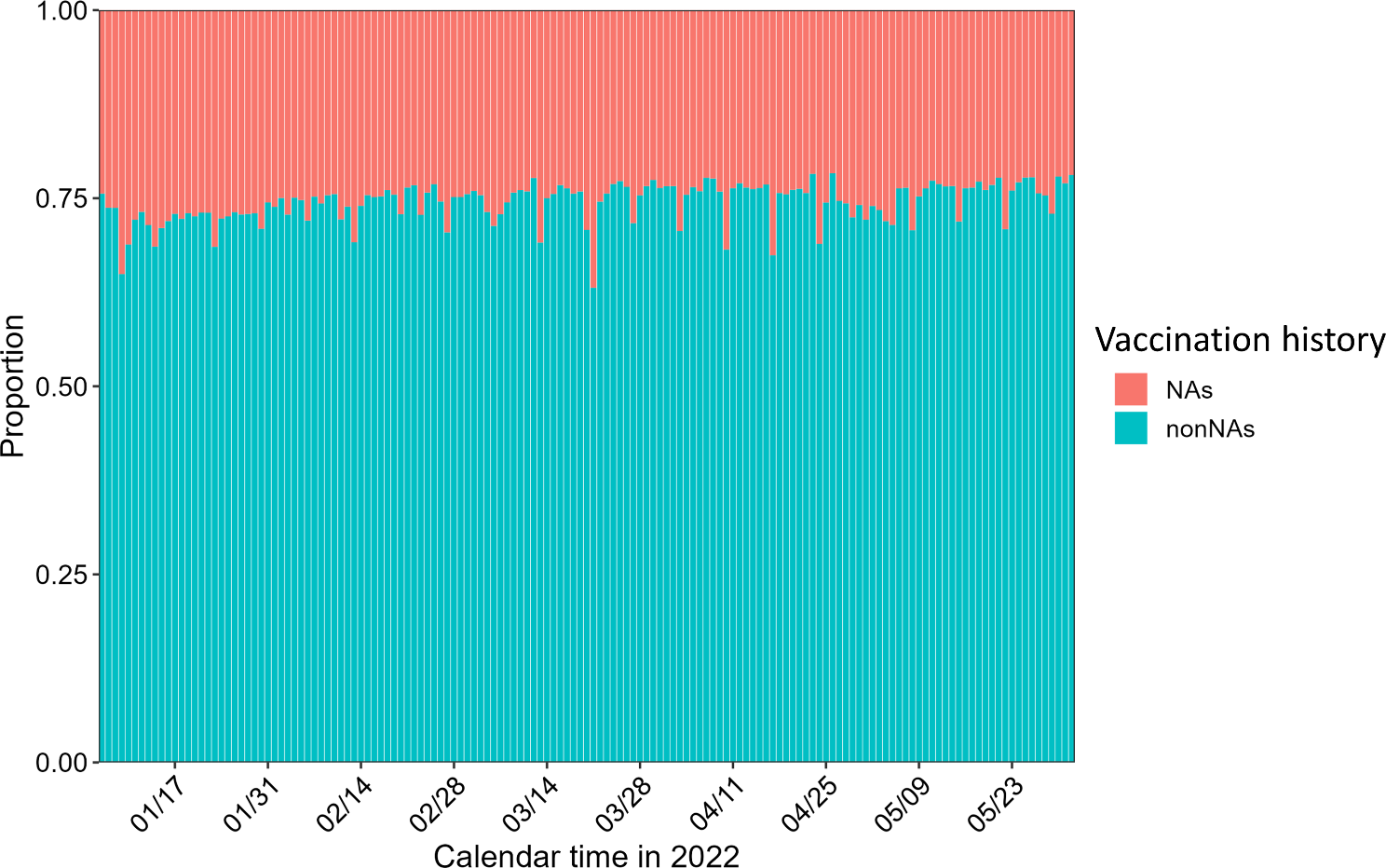


**Figure S5. Proportion of confirmed cases with unknown vaccination history.**

NA denotes "Not available", indicating that the vaccination history remained unknown. Conversely, "nonNA" means that the vaccination history was available.


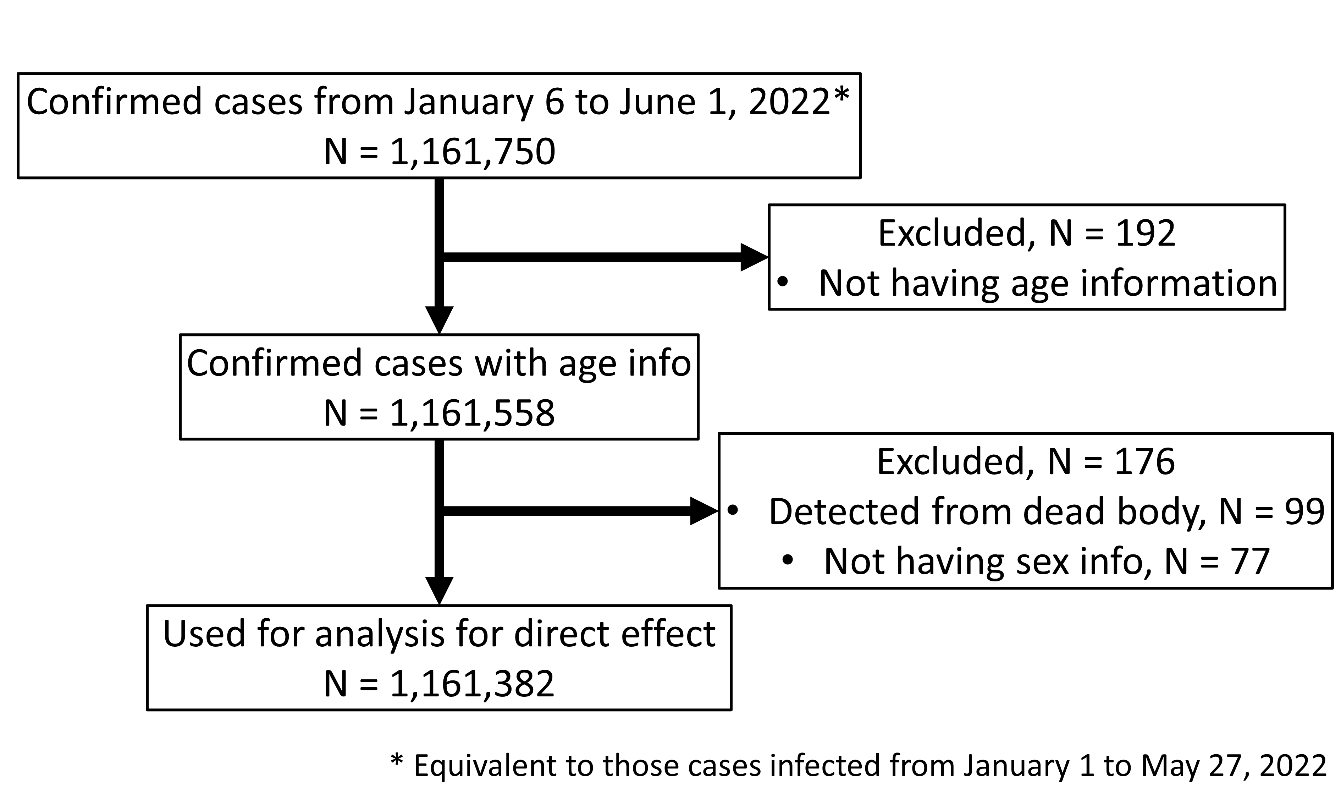


**Figure S6. Flowchart of extracting COVID-19 cases for the analysis.**


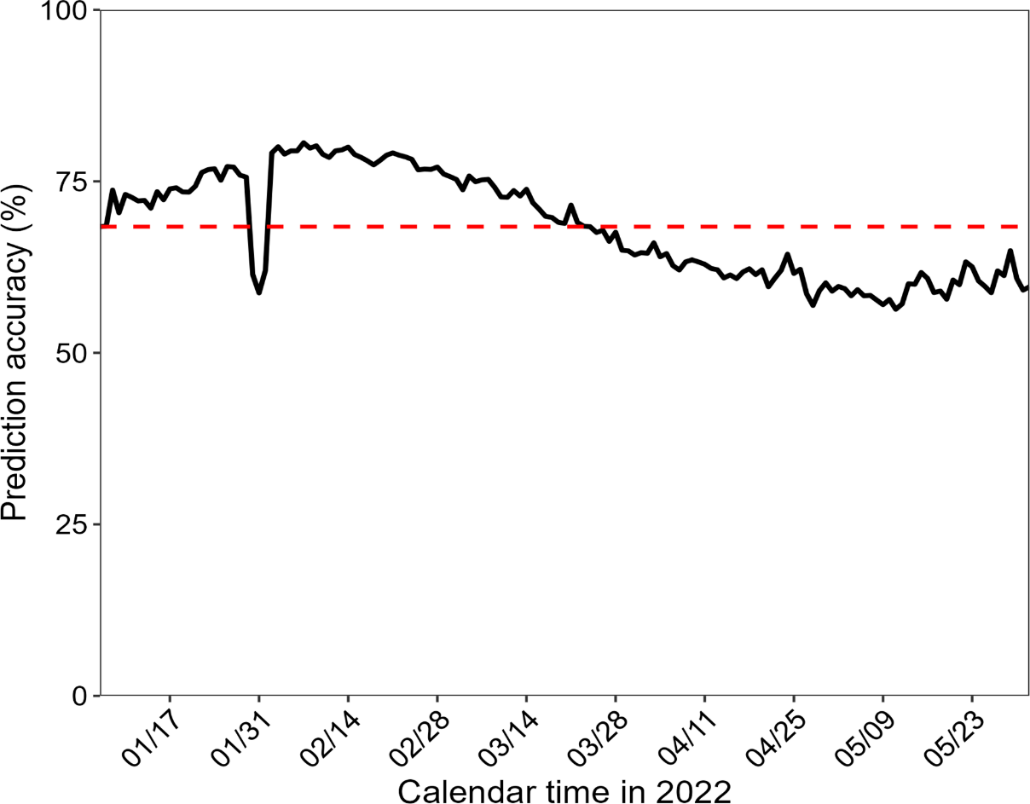


**Figure S7.** **Prediction accuracy of the deterministic approach to impute missing values for vaccination history.**

The dashed red line represents the median prediction accuracy, estimated to be 68%.


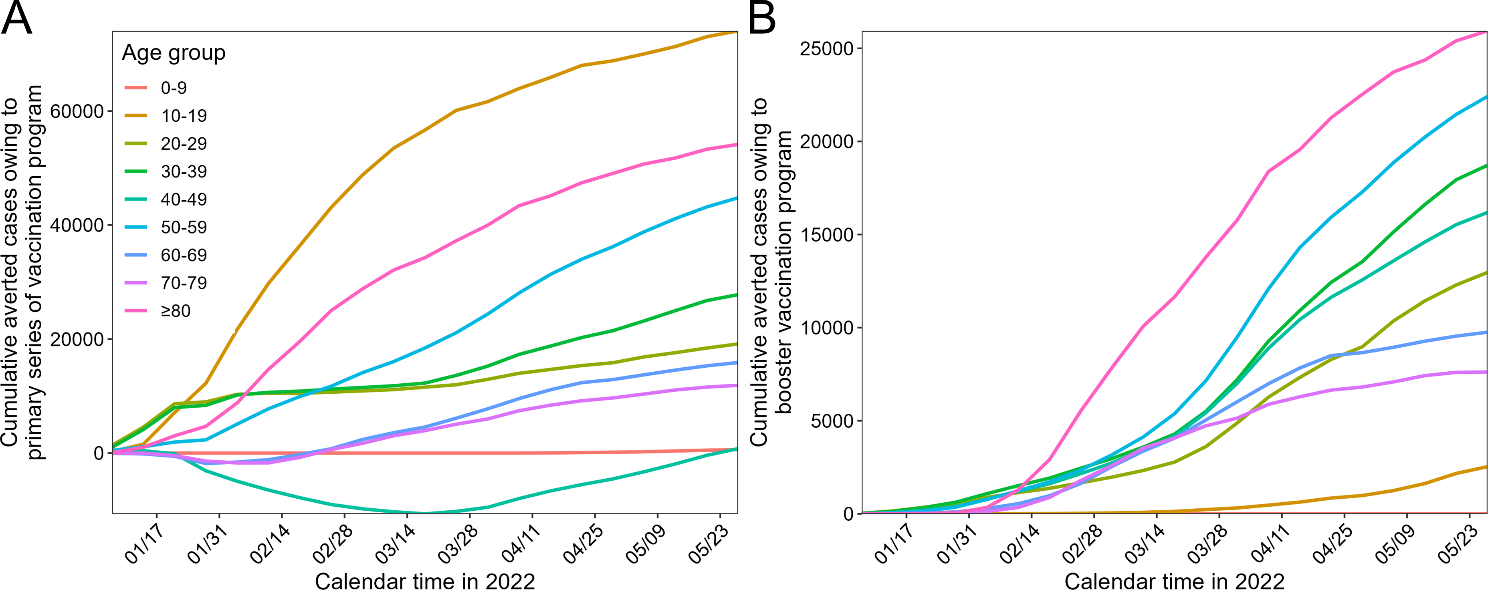


**Figure S8.** C**umulative number of prevented COVID-19 cases based on the deterministic approach.**

Cumulative number of prevented COVID-19 cases directly attributable to (A) the primary series and (B) the booster vaccination program by age group. The number of prevented cases was based on the deterministic approach.


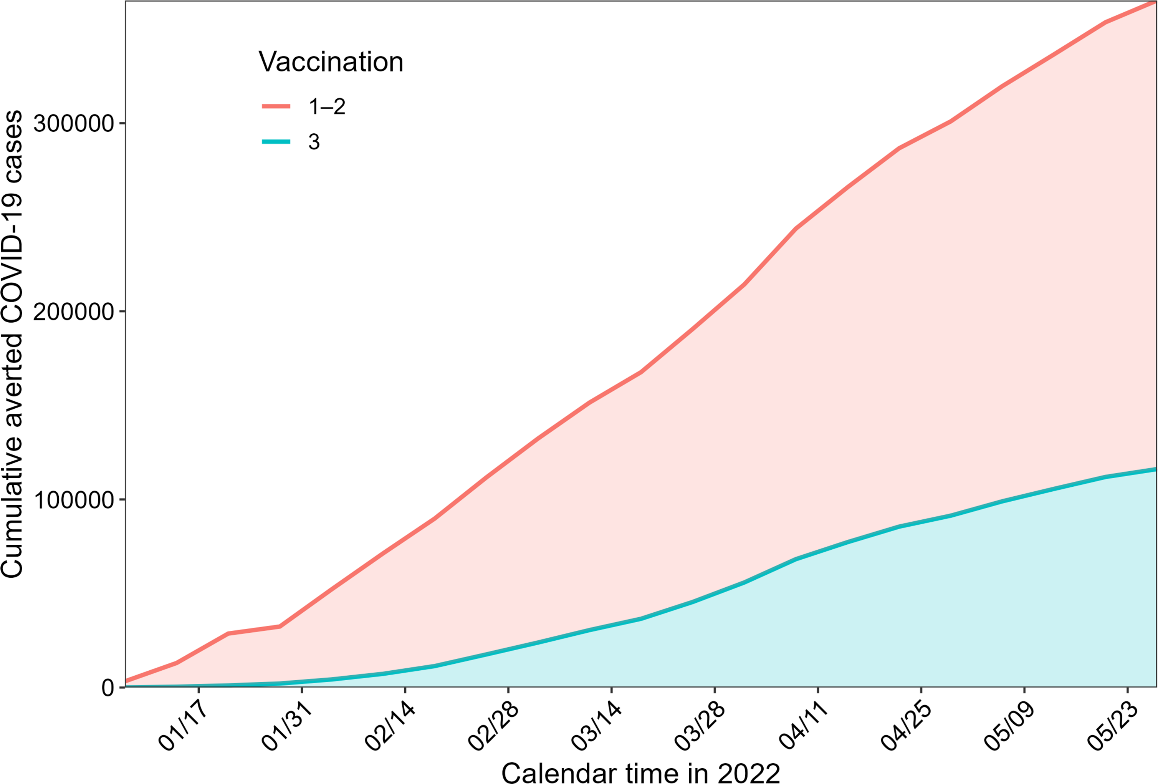


**Figure S9.** **Total number of prevented COVID-19 cases based on the deterministic approach.**

Total number of prevented COVID-19 cases directly attributable to the primary series (1–2 doses) and the booster program (3rd dose). The number of prevented cases was based on the deterministic approach.


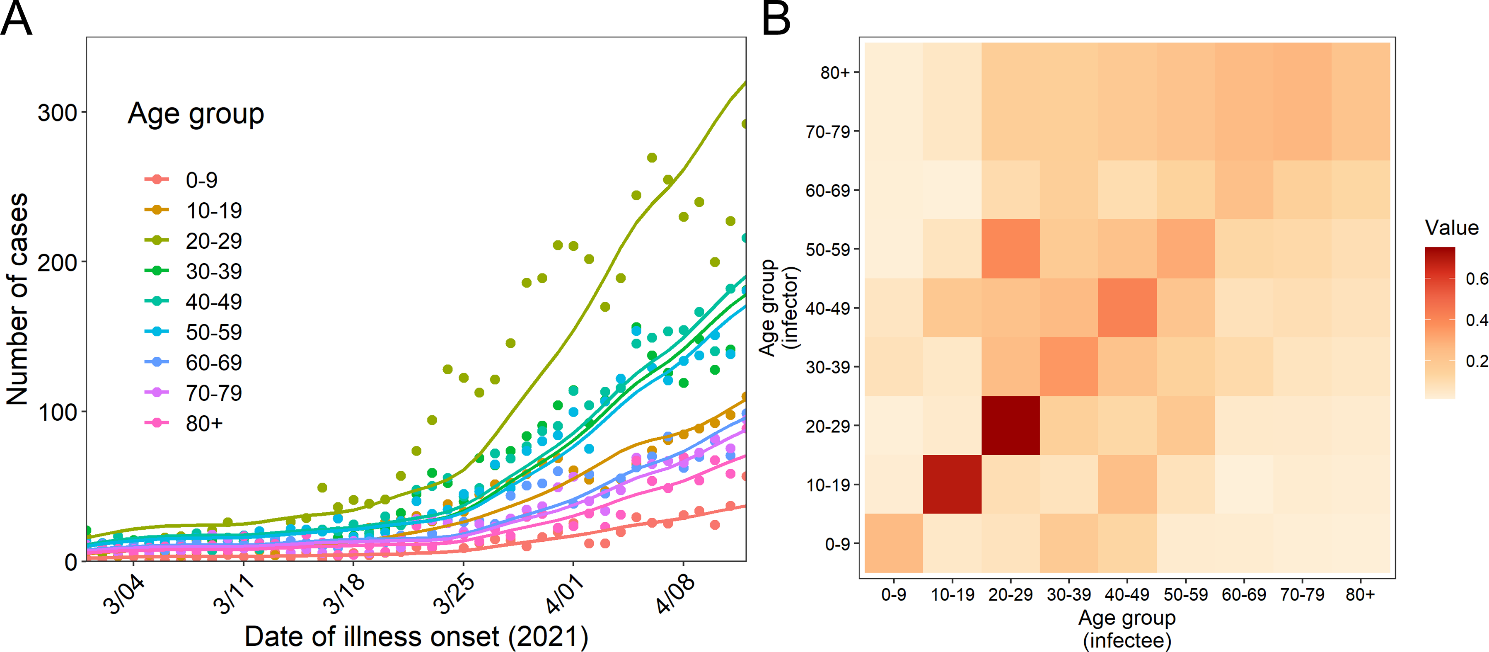


**Figure S10.** **Next-generation matrix.**

The next-generation matrix was estimated in the initial phase of the epidemic due to the Alpha variant in Osaka in March–April 2021. (A) Comparison of predicted and observed cases in Osaka, Japan. Colored dots represent observed cases, and lines represent predicted cases from March 1 to April 12, 2021. (B) Next-generation matrix. The matrix maps the distribution of secondary cases by age group caused by a typical single primary case during the initial phase of an epidemic of Alpha variant in Osaka. The matrix was originally used to compute the effective reproduction number of the Alpha variant as the dominant eigenvalue.


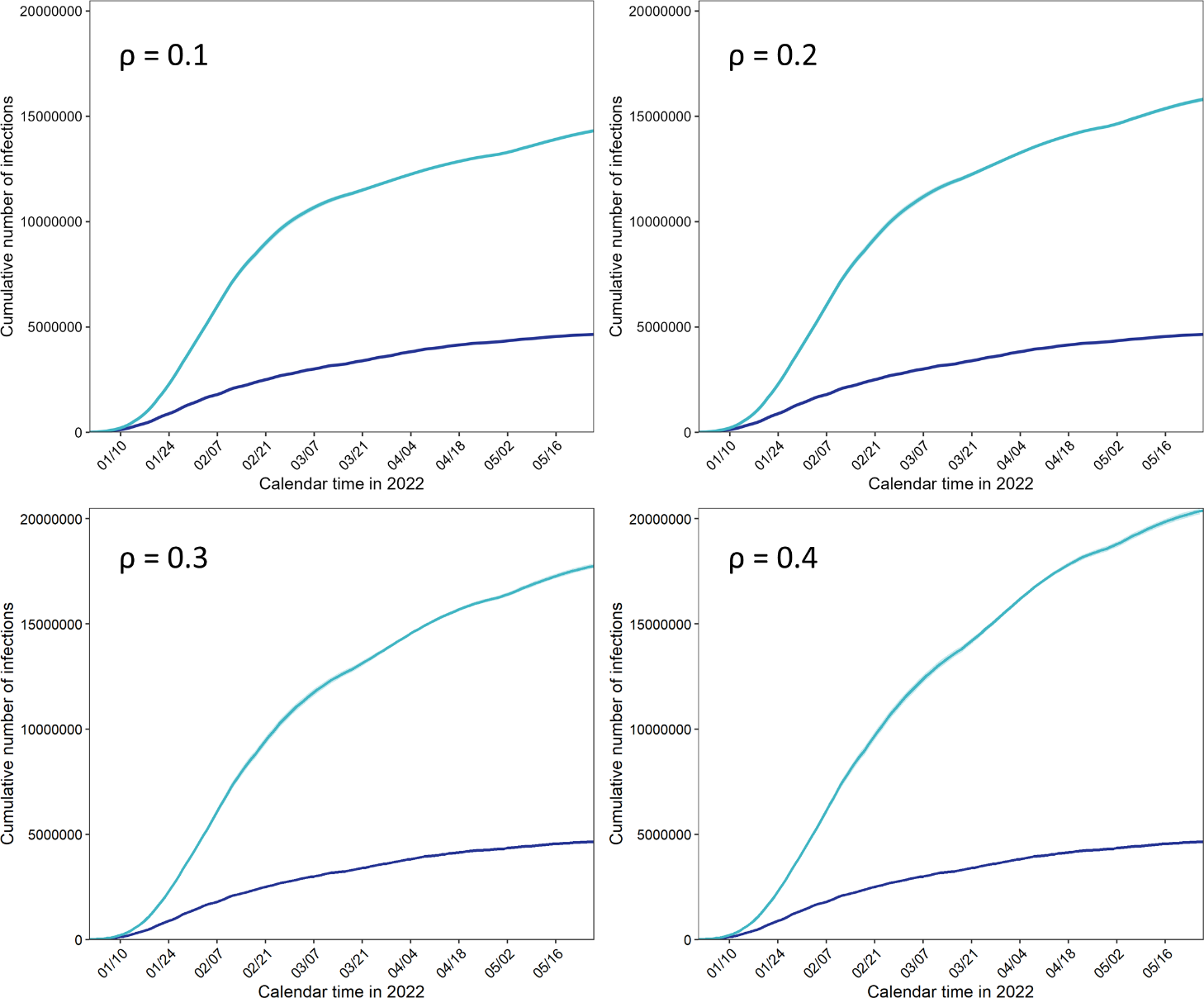


**Figure S11. Cumulative number of SARS-CoV-2 infections: a sensitivity analysis to breakthrough infections.**

The light blue line represents the cumulative number of infections in the counterfactual scenario in which the primary series and booster programs had not taken place. The dark blue line represents the cumulative number of observed infections assuming that the reporting coverage was 25%. $\rho$ represents the history of past vaccination shown in Eq. (6).


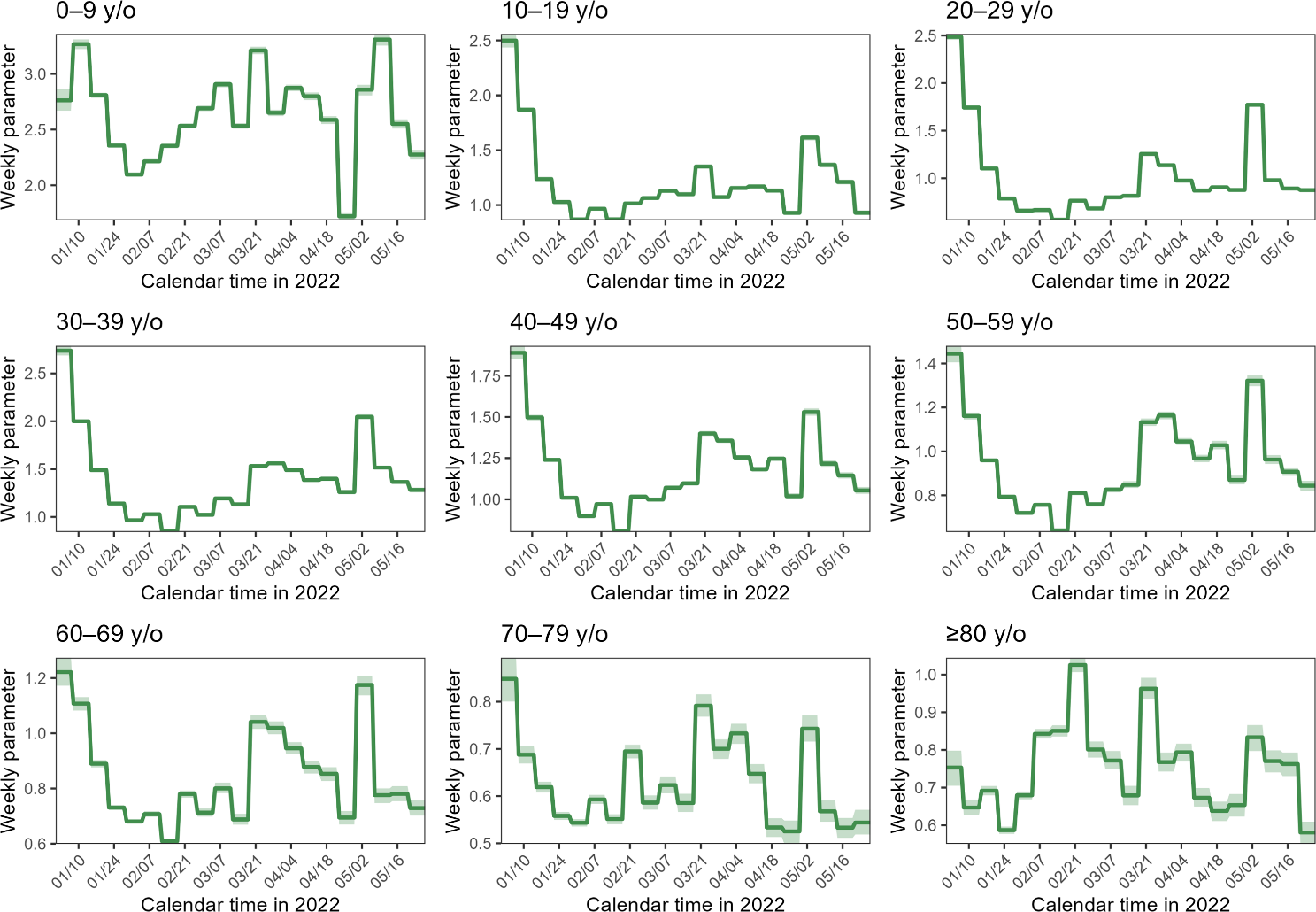


**Figure S12.** **Weekly parameter by age group.**

Weekly parameter ($q_{a,w}$) with 95% confidence interval (light green area) for each week from January 1 to May 27, 2022 by age group.


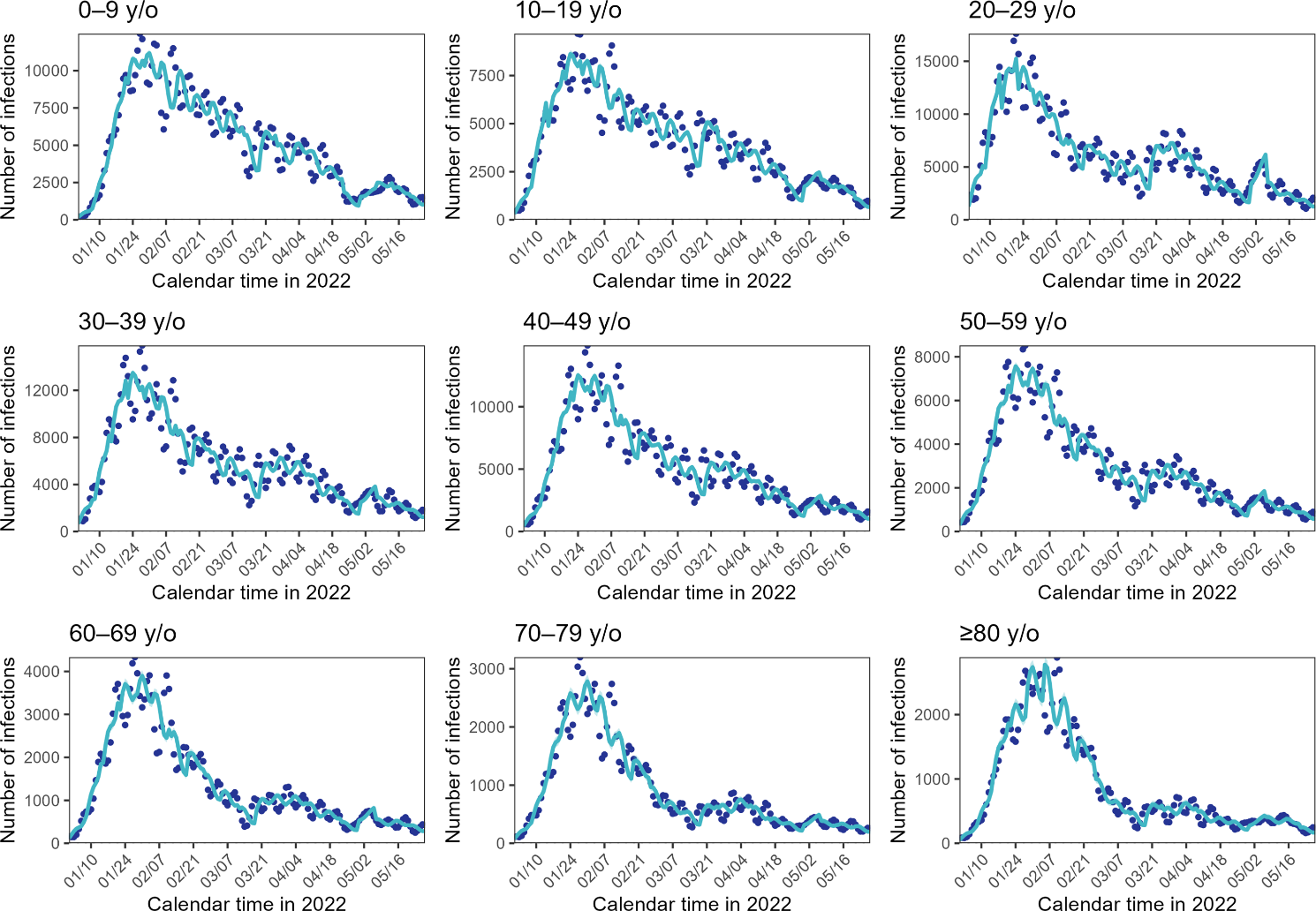


**Figure S13.** **Comparison between observed and predicted SARS-CoV-2 infections by age group.**

Dots represent the number of observed infections given a reporting coverage of 0.25, and each line represents the number of infections predicted by the transmission model.


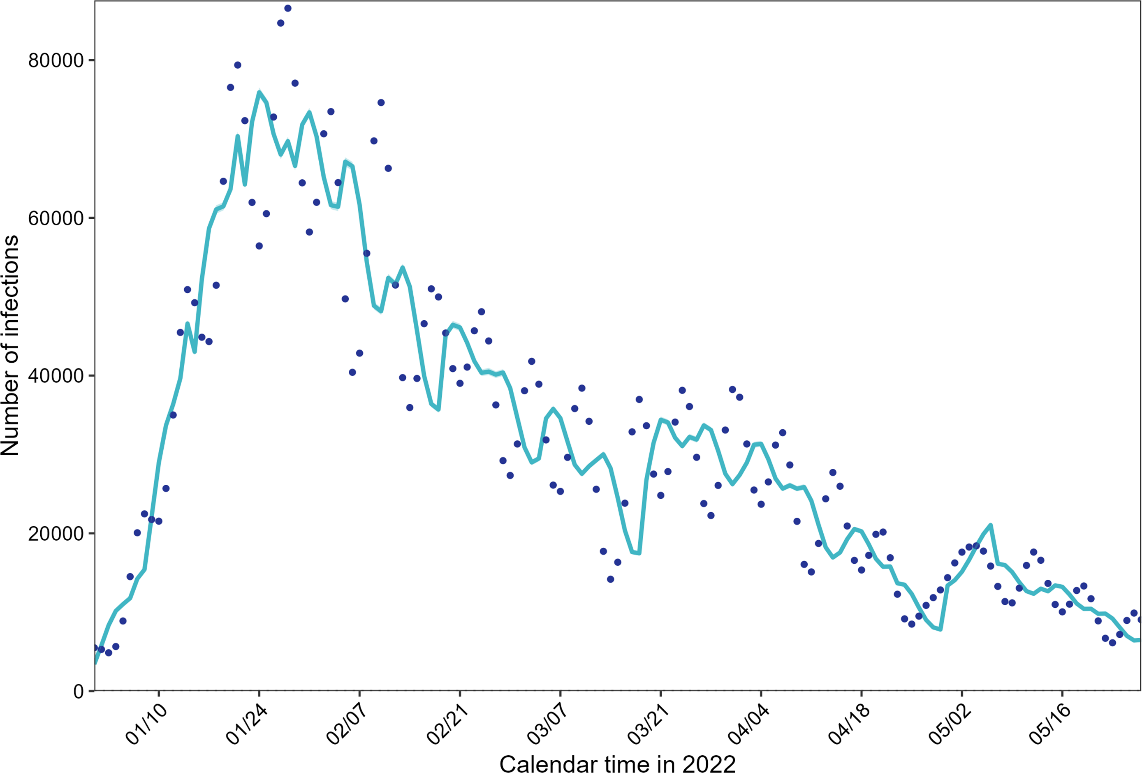


**Figure S14.** **Comparison of the total number of observed and predicted SARS-CoV-2 infections assuming a reporting coverage is 0.25.**

Dots represent the number of observed infections given a reporting coverage of 0.25, and a line represents the number of infections predicted by the transmission model. Note that the vertical axis should be multiplied by 1/0.125 or 1/0.5 to assume that the reporting rate is 0.125 or 0.5, respectively.


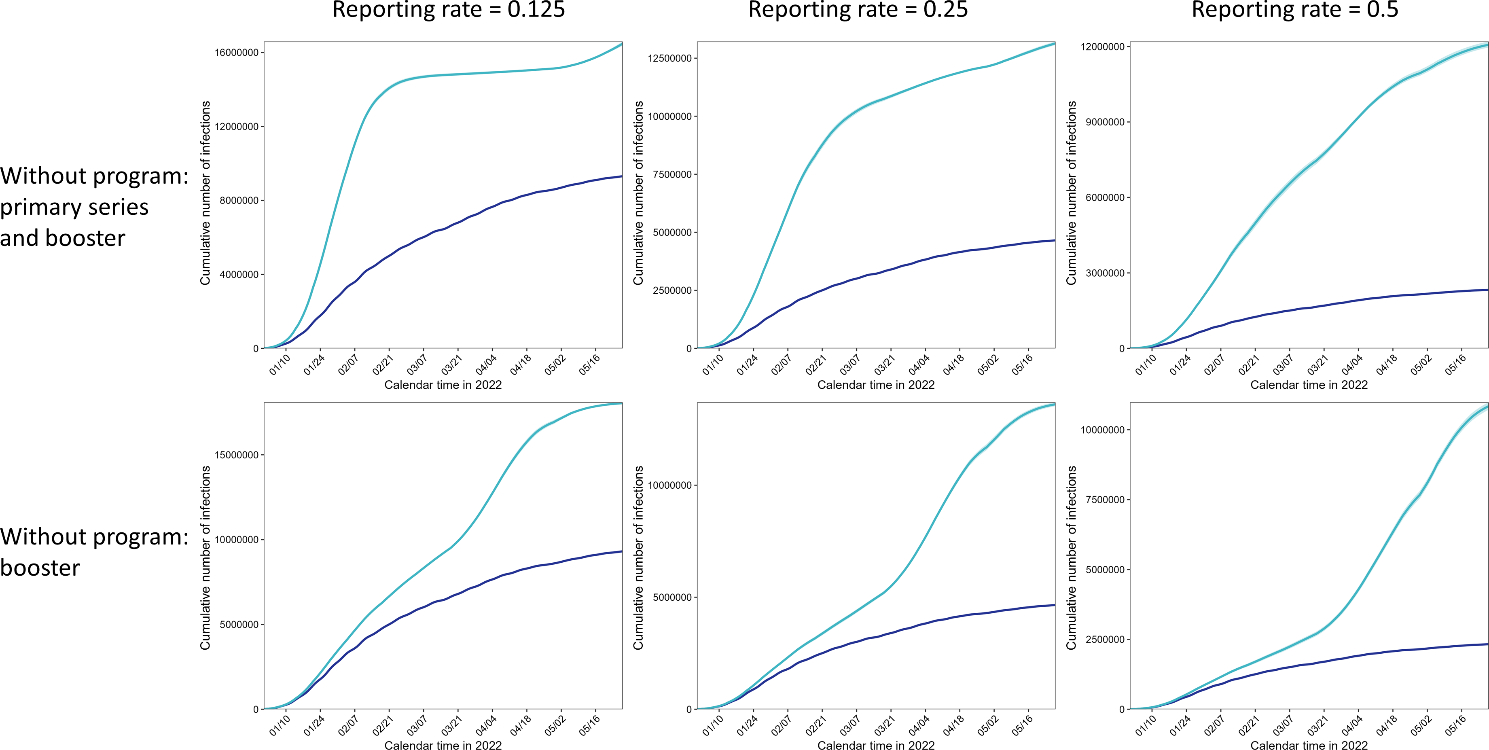


**Figure S15.** **Cumulative number of SARS-CoV-2 infections by reporting coverage.**

The light blue lines represent the cumulative number of infections in the counterfactual scenario in which the primary series and booster programs had not taken place. The dark blue lines indicate the cumulative number of observed infections considering each reporting coverage. Note that the scale of the vertical axis differs.


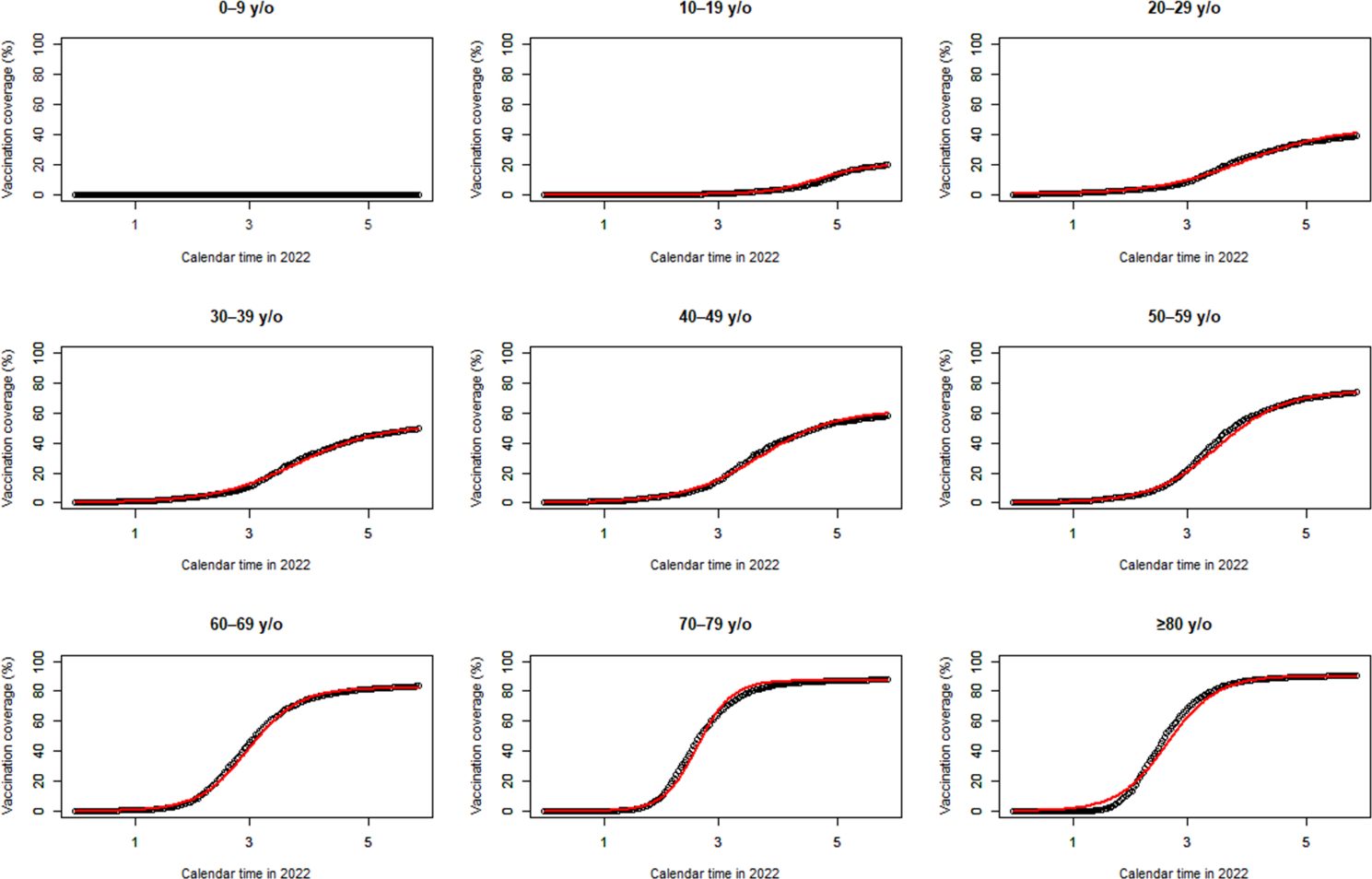


**Figure S16.** **Comparison between observed and predicted vaccination coverage of the booster program by age group.**

Black dots represent the observed vaccination coverage of the booster program. Red lines represent the predicted vaccination coverage using the logistic function.


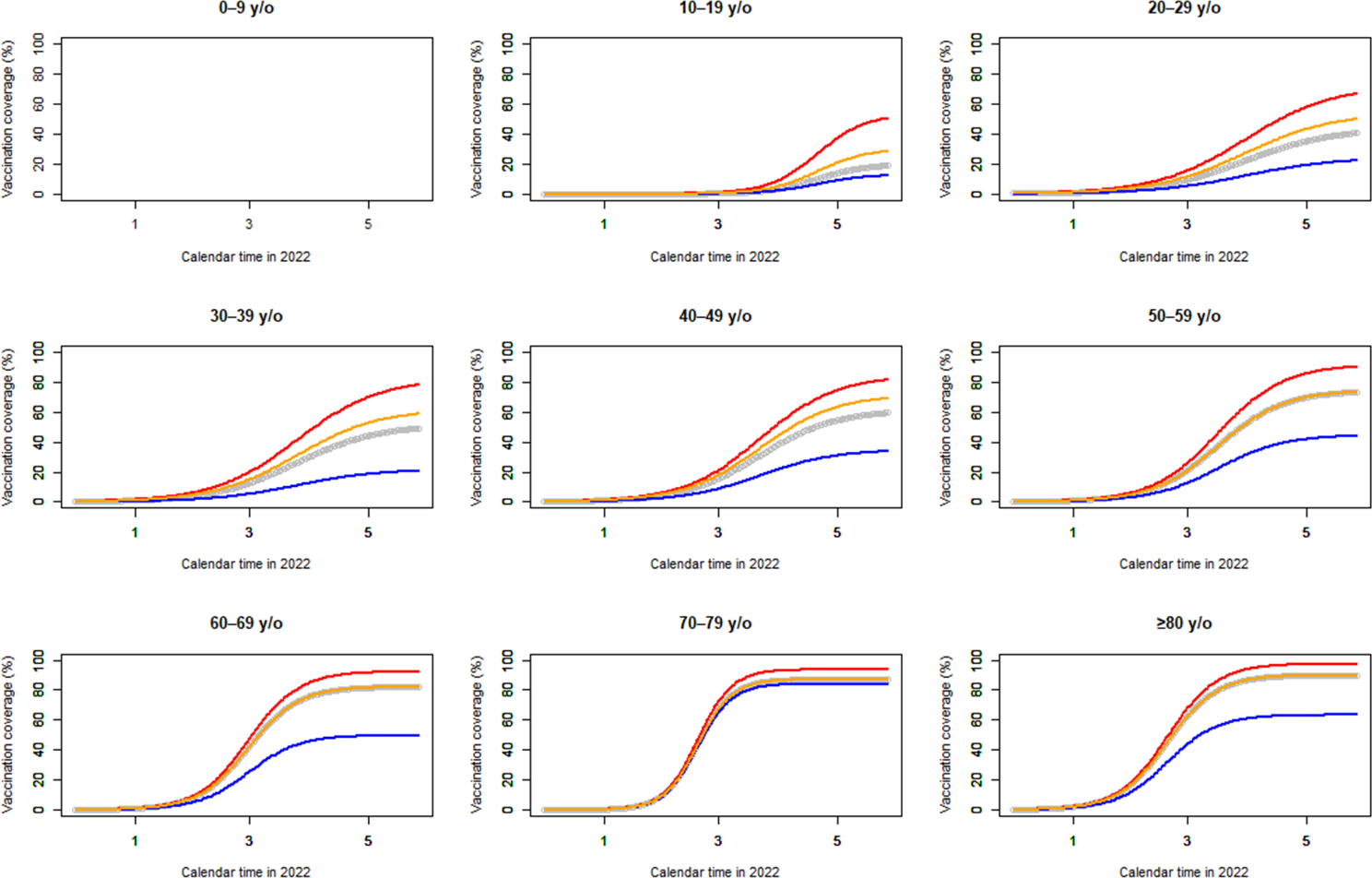


**Figure S17.** **Counterfactual scenarios of the booster vaccination coverage by age group.**

Gray dots represent the baseline scenario vaccination coverage, i.e., the fitted predictions. Red, blue, and orange lines indicate the “Equiv. to 2nd dose” vaccination scenario, the “Equiv. to 4th dose” vaccination scenario, and the “Elevated coverage” vaccination scenario, respectively.

**Supplementary Tables**

**Table S1. Cumulative number of SARS-CoV-2 infections in the absence of vaccination by reporting coverage.**

| Reporting coverage | Without vaccination program^a^ | Model estimate (95% CI^b^) |
| --- | --- | --- |
| 0.125 | Primary series and booster | 16,477,289 (16,370,357–16,591,756) |
| 0.125 | Booster | 18,040,233 (17,993,424–18,09,0792) |
| 0.25 | Primary series and booster | 13,139,409 (13,065,120–13,219,410) |
| 0.25 | Booster | 13,613,769 (13,531,719–13,698,578) |
| 0.5 | Primary series and booster | 10,843,208 (10,714,349–10,978,463) |
| 0.5 | Booster | 12,071,395 (11,962,373–12,198,518) |

^a^ “Primary series and booster” represents the vaccination program for the first, second, and third dose, and “Booster” represents the vaccination program for the third dose.

^b^ Confidence intervals were calculated based on the parametric bootstrap method.

**Table S2. Comparison of population impact of vaccination at the end of the study period (May 27, 2022).**

| Program in absence | Total effect | Direct effect | Indirect effect | Proportion of indirect effect out of total effect (%) |
| --- | --- | --- | --- | --- |
| Primary series and booster | 8,484,581 | 2,558,411 | 5,926,170 | 69.8 |
| Booster | 8,958,941 | 646,326 | 8,312,615 | 92.8 |
